# Enhanced visible light absorption in layered Cs_3_Bi_2_Br_9_ through mixed-valence Sn(ii)/Sn(iv) doping†

**DOI:** 10.1039/d1sc03775g

**Published:** 2021-10-05

**Authors:** Chantalle J. Krajewska, Seán R. Kavanagh, Lina Zhang, Dominik J. Kubicki, Krishanu Dey, Krzysztof Gałkowski, Clare P. Grey, Samuel D. Stranks, Aron Walsh, David O. Scanlon, Robert G. Palgrave

**Affiliations:** Department of Chemistry, University College London 20 Gordon Street London WC1H 0AJ UK r.palgrave@ucl.ac.uk; Thomas Young Centre, University College London Gower Street London WC1E 6BT UK; Department of Materials, Imperial College London Exhibition Road London SW72AZ UK; Cavendish Laboratory, University of Cambridge JJ Thomson Avenue Cambridge CB3 0HE UK; Institute of Physics, Faculty of Physics, Astronomy and Informatics, Nicolaus Copernicus University 87-100 Toruń Poland; Department of Experimental Physics, Faculty of Fundamental Problems of Technology, Wroclaw University of Science and Technology 50-370 Wroclaw Poland; Department of Chemical Engineering & Biotechnology, University of Cambridge Philippa Fawcett Drive Cambridge CB3 0AS UK; Department of Materials Science and Engineering, Yonsei University Seoul 03722 Korea; Diamond Light Source Ltd. Diamond House, Harwell Science and Innovation Campus, Didcot Oxfordshire OX11 0DE UK; Department of Chemistry, University of Cambridge Lensfield Road Cambridge CB2 1EW UK

## Abstract

Lead-free halides with perovskite-related structures, such as the vacancy-ordered perovskite Cs_3_Bi_2_Br_9_, are of interest for photovoltaic and optoelectronic applications. We find that addition of SnBr_2_ to the solution-phase synthesis of Cs_3_Bi_2_Br_9_ leads to substitution of up to 7% of the Bi(iii) ions by equal quantities of Sn(ii) and Sn(iv). The nature of the substitutional defects was studied by X-ray diffraction, ^133^Cs and ^119^Sn solid state NMR, X-ray photoelectron spectroscopy and density functional theory calculations. The resulting mixed-valence compounds show intense visible and near infrared absorption due to intervalence charge transfer, as well as electronic transitions to and from localised Sn-based states within the band gap. Sn(ii) and Sn(iv) defects preferentially occupy neighbouring B-cation sites, forming a double-substitution complex. Unusually for a Sn(ii) compound, the material shows minimal changes in optical and structural properties after 12 months storage in air. Our calculations suggest the stabilisation of Sn(ii) within the double substitution complex contributes to this unusual stability. These results expand upon research on inorganic mixed-valent halides to a new, layered structure, and offer insights into the tuning, doping mechanisms, and structure–property relationships of lead-free vacancy-ordered perovskite structures.

## Abstract 2

Mixed valence Sn doping of Cs_3_Bi_2_Br_9_ leads to broad visible light absorption.

## Graphical abstract

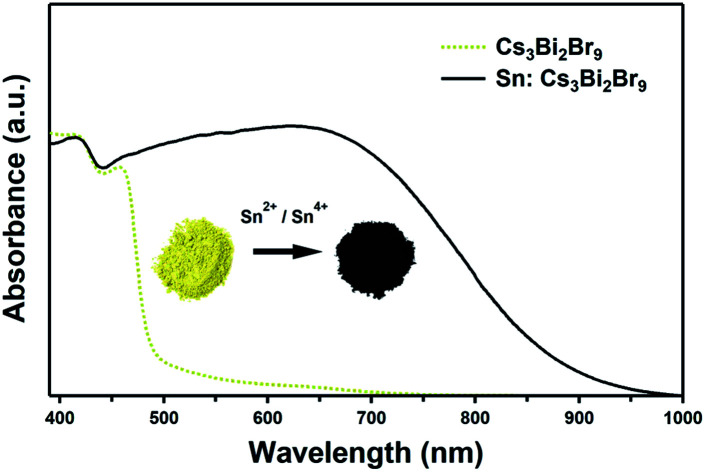

## Introduction

Metal halides that form the perovskite structure with composition ABX_3_, where A is a group 1 metal or molecular organic cation, B is a divalent cation, and X is a halide anion, have been intensely studied for their exceptional optoelectronic properties.^1^ Concerns over instability and toxicity of a commonly used B(ii) cation, Pb, have led to exploration of alternative ternary metal halides with structures closely related to the perovskites.^2^ The cubic A_2_BX_6_ structure, commonly called the vacancy-ordered double perovskite structure,^3^ consists of B(iv) cations such as Sn, Te, Ti, Pt *etc.*, while the composition A_3_B_2_X_9_ contains B(iii) cations such as Sb and Bi, and forms several different crystal structures with varying degrees of connectivity in the B–X sublattice.^4–10^ The large majority of reported A_3_B_2_X_9_ compounds adopt a hexagonal structure in the space group *P*6_3_/*mmc*.^11,12^ This structure can be thought of as hexagonal close packed A_3_X_9_ with B cations ordered in octahedral interstitial sites, resulting in isolated [B_2_X_9_]^3−^ dimers, an arrangement often described as zero dimensional (0D). Two other main structure types are known, each adopted by only a handful of reported compositions: infinite one-dimensional (1D) double chains based on corner-sharing [BX_6_]^3−^ octahedra,^13,14^ and two-dimensional (2D) corrugated layers featuring corner-connected [BX_6_]^3−^ octahedra.^8,15–19^ Of these, the 2D layered structure is most closely related to the cubic perovskites, and can be described as a vacancy-ordered triple perovskite, formed by removing every third B site cation from cubic ABX_3_. The resulting local B cation coordination environment is irregular and exhibits a trigonal distortion that produces three short and three long B–X bonds, with the shorter linkages shared between neighbouring octahedra in each layer.^9^ Both theoretical and experimental investigations have found that for a given composition, this 2D layered A_3_B_2_X_9_ polymorph exhibits a narrower band gap, higher electron and hole mobility, larger dielectric constants, and corresponding better defect tolerance,^20,21^ as well as enhanced stability towards degradation, compared with the other A_3_B_2_X_9_ structures.^22^ This likely renders the layered form more suitable than the 1D chain or 0D dimer counterparts for optoelectronic applications including photovoltaics and lighting. However, the 2D layered structure only forms for a few compositions, most commonly bromides and chlorides such as Cs_3_Bi_2_Br_9_, Cs_3_Sb_2_Br_9_ and Cs_3_Fe_2_Cl_9_. The band gaps of these compounds are relatively wide due to the high electronegativity of the halogen. While the 2D structure has inherent benefits, its stabilisation only by lighter halides means the band gaps of 2D compounds are typically large. Therefore, whilst developing methods to control the band gap and enhance optical absorption in lead-free halide materials is of general concern, it is especially important for 2D A_3_B_2_X_9_ compounds.

The wide range of ions that can be incorporated on both the cation and anion sites of an A_3_B_2_X_9_ compound allows both the crystal and electronic structure to be optimised by judicious choice of composition.^2^ Several strategies have been employed, from alloying on the halide site,^8,23–25^ which usually produces a band gap response similar to that seen in the perovskites, to doping on the B-metal site, which may lead to introduction of filled states within the band gap,^26,27^ or band bowing.^26–28^

Recently, Ghosh *et al.* studied a mixed valence perovskite (CH_3_NH_3_)AuBr_3_, containing Au(i) and Au(iii), which resulted in significant visible light absorption.^29^ They suggest that mixed valency may be a way to improve the optical response for photovoltaic or other applications. Mixed-valence compounds, those containing a single element in multiple oxidation states, are known to display optical and electronic transport properties not seen in analogous single-valence compounds. Mixed-valence oxides have very diverse properties, such as superconductivity and magnetic ordering,^30,31^ while mixed-valence halides have been studied less extensively. McCall *et al.* recently reported the properties of CsInX_3_ (X = Br, Cl), mixed valence indium compounds which similarly show intense visible light absorption.^32^ Other examples of mixed valence compounds that form perovskite-like structures are CsAuCl_3_ and CsAgCl_3_.^33,34^ However, past work on mixed-valence halides has focused almost exclusively on cubic A_2_BX_6_ compounds. For example, Cs_2_SbCl_6_ consists of equal amounts of Sb(v) and Sb(iii), and displays an intense blue colour and high conductivity, compared with Cs_2_SnCl_6_, formally containing only Sn(iv), which is colourless and insulating.^35–37^ The blue colour is ascribed to a single very strong absorption band near 2.3 eV, arising from a transition in which an electron is transferred from the highest occupied orbital of the lower oxidation state ion to the lowest unoccupied orbital in the higher oxidation state ion, *i.e.*, an intervalence charge transfer (IVCT). Forming solid solutions of these two compounds (Cs_2_Sb_*x*_Sn_1−*x*_Cl_6_) results in materials with colours ranging from an intense dark blue through to green, yellow, and finally to colourless, as *x* goes from 1 to 0. Despite the intriguing optoelectronic properties conferred, the field of mixed-valence doping in inorganic halides remains largely unexplored beyond the foundational work on the cubic A_2_BX_6_ compounds and more recent work on ABX_3_ perovskites.

As outlined above, vacancy-ordered triple perovskites of the A_3_B_2_X_9_ type provide a useful arena for study of the effects of crystalline and electronic dimensionality on material properties, in contrast with cubic A_2_BX_6_ compounds which uniformly have isolated BX_6_ octahedra. While the substitution of Sn(iv) with equal amounts of Sb(iii) and Sb(v) has been described in A_2_BX_6_,^38,39^ in this work we study the effects of substitution of Bi(iii) with Sn(ii) and Sn(iv) in A_3_B_2_X_9_. We show that doping Sn into Cs_3_Bi_2_Br_9_ results in a drastic redshift of the optical absorption onset that can be attributed to transformation of the material into a mixed-valent state, which can be described as a Robin–Day compound exhibiting strong IVCT. We characterise a range of materials with different Sn contents using experimental and computational methods. Our results expand knowledge of mixed-valent compounds to a new structure type, and offer insights into the tuning, doping mechanisms, and structure–property relationships of lead-free perovskite-inspired structures.

## Experimental section

Cs_3_Bi_2_Br_9_ was prepared *via* a solution-phase method carried out in air. An acidic solution of CsBr was produced by reacting 1.5 mmol Cs_2_CO_3_ with 2 mL aqueous HBr (3 M). Separately, 2 mmol BiBr_3_ was dissolved in 5 mL aqueous HBr (3 M) and stirred on a heating mantle at 80 °C. After 10 min of stirring, the CsBr solution was added to the BiBr_3_ solution. An immediate yellow precipitate appeared, which was isolated by filtration, washed with ethanol, and stored in air.

Synthesis of Sn doped Cs_3_Bi_2_Br_9_ was carried out by mixing the desired ratio of SnBr_2_ and BiBr_3_ in solution, followed by reaction with acidic aqueous CsBr solution. For example, to obtain a nominal Sn dopant concentration of 10%, 0.22 mmol SnBr_2_ and 2 mmol BiBr_3_ were dissolved by stirring in 5 mL HBr (3 M) at 80 °C, followed by addition of the CsBr solution as described above. Samples with varying nominal amounts of Sn (
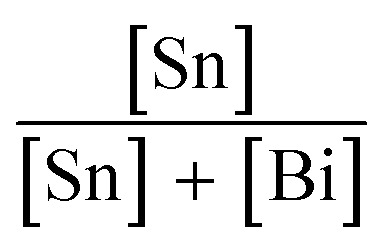
 = 0, 5, 7, 10, 15, 30, 40, and 50%) were synthesised in this way. When Sn was present, a dark brown or black precipitate was formed, depending on the Sn concentration. The product was isolated by filtration, washed with ethanol, and stored in air. Note that the only species of tin added to the reaction was Sn(ii). If the reaction was attempted with SnBr_4_ instead of SnBr_2_, a yellow product was formed at all Sn concentrations.

X-ray photoelectron spectroscopy measurements were carried out in a Thermo K-alpha spectrometer utilising a 72 W monochromated Al K-α X-ray source (*E* = 1486.6 eV) and focused to a spot of 400 μm diameter at the sample surface. Powder X-ray diffraction was measured using a STOE Stadi P diffractometer equipped with a STOE Dectris Mythen 1K detector and operating with Mo Kα radiation (*λ* = 0.70930 Å) in transmission mode. UV-Vis spectra were recorded in diffuse reflectance mode using a Shimadzu UV-2600 spectrometer equipped with an integrating sphere (Shimadzu ISR-2600). The reflecting reference used was a barium sulfate pellet. Samples were diluted with barium sulfate powder for measurement; spectra taken with different concentrations of sample to barium sulfate showed no significant differences (ESI Fig. S16†). Reflectivity (*R*) data were converted using the Kubelka–Munk relationship: *F*(*R*) = (1 − *R*)^2^/2*R*. Room temperature ^133^Cs (52.5 MHz) and ^119^Sn (149.4 MHz) MAS NMR spectra were recorded on a Bruker Avance Neo 9.4 T spectrometer equipped with a 4.0 mm CPMAS probe. ^133^Cs shifts were referenced using solid CsI as a secondary reference (*δ* = 271.1 ppm). ^119^Sn spectra were referenced using solid SnO_2_ (^119^Sn *δ* = −603.4 ppm). Calibrated RF strengths: ^133^Cs (62.5 kHz), ^119^Sn (125 kHz). The ^119^Sn spectra were acquired chunk-wise owing to the large chemical shift range of halostannates.^40^ The quantitative recycle delays were set on the basis of the measured *T*_1_ values, as detailed in Table S2.† All solid-state experiments were carried out at 12 kHz MAS. Further experimental details are given in the Table S2.† Photoluminescence (PL) spectra were obtained using a fixed grating Maya 2000 Pro spectrometer. A He flow vacuum cryostat was used to achieve a temperature of 10 K. Samples were excited using a continuous wave 405 nm laser excitation source operating at low power density (∼20 mW cm^−2^). To ensure thermal contact, thin layers of powder were placed between cooling plates (copper/sapphire glass).

## Computational methodology

All calculations were performed using Density Functional Theory (DFT) within periodic boundary conditions through the Vienna *Ab Initio* Simulation Package (VASP).^41–45^ The range-separated, screened hybrid DFT exchange–correlation functional of Heyd, Scuseria and Ernzerhof with 25% exact Hartree–Fock exchange (HSE06) was used for geometry optimizations, calculations of optical dielectric constant, total energies and electronic band structures.^46^ To fully account for relativistic effects, spin–orbit interactions were included (HSE06 + SOC) in all total energy, electronic and optical calculations. This level of theory is found to accurately reproduce the experimental electronic structure of Cs_3_Bi_2_Br_9_, an absolute necessity for accurate prediction of defect properties.^47,48^ Using the projector-augmented wave method, scalar-relativistic pseudopotentials were employed to describe the interaction between core and valence electrons.^49^ The optical dielectric response was calculated using the method of Furthmüller *et al.*^50^ to obtain the high-frequency real and imaginary dielectric functions.

A convergence criterion of 0.01 eV Å^−1^ was imposed on the forces on each atom during structural optimization, both for bulk material and defect supercells. Bulk electronic structure calculations were carried out with a 14-atom primitive unit cell, using a well-converged 4 × 4 × 3 *Γ*-centred Monkhorst–Pack *k*-point mesh (equivalent to a *k*-point density of 0.22 Å^−1^) and a 350 eV plane-wave energy cutoff. To ensure accurate structural parameters and to avoid the possibility of spurious Pulay stress,^51^ a higher plane-wave energy cutoff (500 eV) and a denser *k*-point mesh (5 × 5 × 4) were employed for geometry optimisation of the primitive bulk. In each calculation, convergence with respect to *k*-point density and plane-wave energy cutoff was confirmed for the property of interest.

Further computational details are given in the ESI.†

## Results

Cs_3_Bi_2_Br_9_ powders were synthesised *via* the solution phase method described above, with the addition of SnBr_2_ in order to give nominal Sn doping levels of 0, 5, 7, 10, 15, 30, 40, and 50%. X-ray photoelectron spectroscopy (XPS) was used to determine the analytical Sn concentrations in these powder samples. Although XPS is a surface technique, we have shown that it gives an accurate reflection of the bulk composition in similar low temperature solution-phase processed halides.^52^ The results for analytical Sn concentrations are summarised in Fig. S1 (ESI†). At low nominal Sn concentrations, the analytical concentration is approximately linear with respect to the nominal amounts used, only plateauing at analytical concentrations of around 20%. The highest analytical Sn level observed was 23.5%; this with a nominal Sn level of 50% Sn. Above this level of nominal Sn content, a Cs_2_SnBr_6_ secondary phase is clearly observed in powder XRD; samples with greater than 50% nominal Sn content were not studied further. We will denote samples by their analytical Sn content from now onwards, although as will be discussed later, NMR shows that some secondary Sn containing phases are present even in samples where none are detected by powder XRD. Therefore, the Sn content should not be interpreted as entirely on the B site of the Cs_3_Bi_2_Br_9_ structure. This point will be discussed further below.

**Fig. 1:**
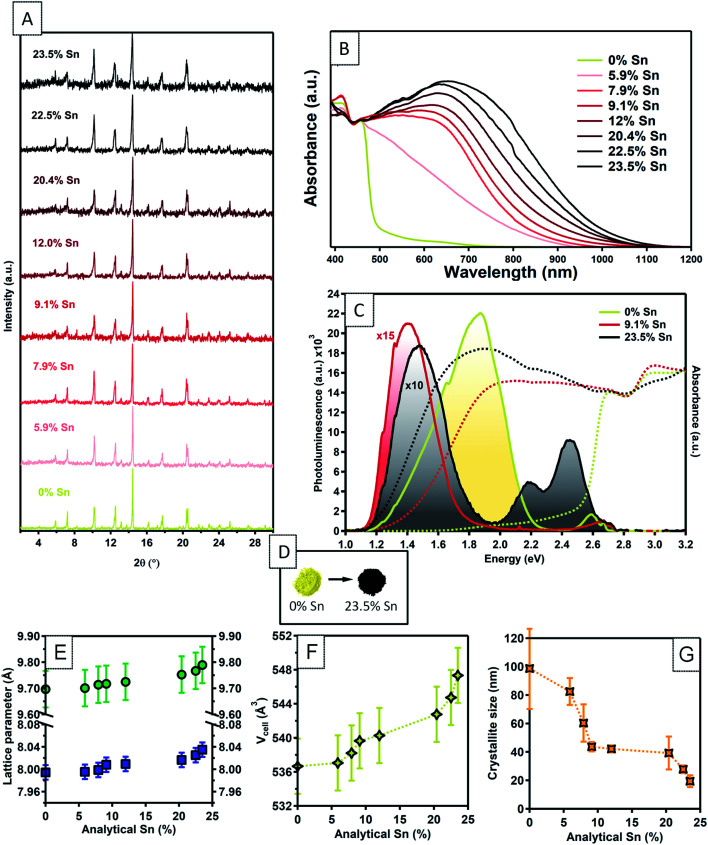
(A) Powder XRD patterns (*λ* = 0.709 Å) from undoped and Sn-doped Cs_3_Bi_2_Br_9_ powders. (B) Absorption spectra obtained after Kubelka–Munk transformation of diffuse reflectance data for the undoped and Sn-doped Cs_3_Bi_2_Br_9_ samples synthesised. (C) Low-temperature (10 K) steady-state photoluminescence spectra (solid lines) obtained for the undoped and Sn-doped Cs_3_Bi_2_Br_9_. Intensities are normalised by the multiplication factors given. Overlayed are the room temperature absorption spectra (dotted lines) of the selected samples. (D) A photograph of undoped and Sn-doped powders. (E) Lattice parameters of Sn-doped Cs_3_Bi_2_Br_9_ derived from powder XRD, blue squares *a* parameter, green squares, *c* parameter. (F) Cell volume of Sn-doped Cs_3_Bi_2_Br_9_ derived from powder XRD. (G) Crystallite size of Sn-doped Cs_3_Bi_2_Br_9_ obtained from a Williamson–Hall plot.

Powder XRD measurements on the as-synthesised samples were consistent with a *P*3̄*m*1 layered vacancy-ordered triple perovskite structure (Fig. 1A).^15,53^

A slight increase in the *a* parameter was observed with increasing Sn concentration, rising from 7.985(5) Å in the undoped material to 8.036(5) Å in the 23.5% Sn doped sample. In contrast, the increase in *c* parameter observed is the same size as the uncertainty in the measurement (Fig. 1E). This suggests that the interlayer spacing is not greatly affected by the presence of Sn, while a small increase in the in-plane distances is seen. Overall, the unit cell volume rises by a total of 2.1% across the doping range investigated here. The full width at half maximum (FWHM) of the powder diffraction peaks increased significantly with increasing doping, almost doubling in the highest doped compound compared with the undoped materials. A Williamson–Hall plot (ESI Fig. S15†) was used to extract the crystallite sizes (assuming negligable peak broadening due to disorder), which decreased from around 100 nm in the undoped material, to below 20 nm for the highest level of doping (Fig. 1G), showing that Sn doping decreased the crystallinity of the material.

The optical absorption of the compounds was studied by diffuse reflectance and photoluminescence spectroscopy (Fig. 1). The absorption profile for undoped Cs_3_Bi_2_Br_9_ is consistent with previous reports,^54^ with the onset of a strong absorption edge at around 430 nm. Tauc analysis yields a band gap of 2.58 eV, very similar to previous measurements of the experimental band gap which range between 2.53 and 2.65 eV,^19,55,56^ and a previous calculated bandgap of 2.64 eV.^19^ Here, we calculate the direct band gap for the host material to be 2.66 eV, using the HSE06 + SOC hybrid DFT functional. With no significant sub band gap absorption, our undoped sample of Cs_3_Bi_2_Br_9_ appeared as a pale yellow powder (Fig. 1D). Sn doping caused very striking changes in the appearance of the samples. Even low amounts of Sn doping led to significant darkening of the powder, and above *ca.* 8% analytical Sn concentration, the powders appeared jet-black (Fig. 1D). The optical spectra of Sn: Cs_3_Bi_2_Br_9_ materials show a very broad, intense absorption reaching across the visible and into the near-infrared region. The absorption peak maximum gradually decreases in energy with increasing Sn content, with absorption maximum at 2.08 eV for 7.9% Sn concentration, moving to 1.90 eV at 23.5% Sn concentration. The significant changes to the optical spectra upon incorporation of Sn is suggestive of a mixed-valence compound, which are known to exhibit strong visible light absorption.^35^ The nature of the Sn dopants was addressed though a combined experimental and theoretical methodology.

We attempted doping using the tetravalent state of tin by replacing the SnBr_2_ used in the synthetic method with SnBr_4_. The resulting powders were bright yellow, and clearly distinguishable from the black colour resulting from synthesis with a molar equivalent amount of SnBr_2_. XRD measurements confirmed that even at low nominal amounts of SnBr_4_, the synthesis results in a biphasic mixture of Cs_2_SnBr_6_ along with Cs_3_Bi_2_Br_9_. Thus, we can confirm the necessity of the divalent tin precursor in this synthesis in order to produce strongly visible light absorbing powders.

The impact of Sn doping on the optical properties of Cs_3_Bi_2_Br_9_ was further investigated by photoluminescence (PL) spectroscopy (Fig. 1E). Spectra were obtained at low temperature (10 K) due to a lack of measurable PL signal at room temperature. Undoped Cs_3_Bi_2_Br_9_ reveals two distinctive blue and red emission branches, reproducing findings from an earlier study.^57^ At 10 K, the photoluminescence spectrum is dominated by the red branch, consisting of a main peak centred at around 1.85 eV, with a tail expanding towards lower energies, which is attributed to emission from bismuth centres near impurities.^57^ The weaker blue emission consists of a narrow free exciton peak followed by emission from shallow defects. Two Sn doped samples were studied, with Sn concentrations of 10% and 23.5%. At both Sn concentrations studied, the emission at 1.85 eV disappears and is replaced by a redshifted peak (centred at 1.40 eV for Sn: 10%, and 1.48 eV for Sn: 23.5%) with a shape closely resembling a mono-Gaussian distribution. Significant changes in the high energy range for the spectrum of 23.5% Sn: Cs_3_Bi_2_Br_9_ are also observed, with an emergence of two relatively strong green emission peaks. Whilst the particular optical transitions leading to the additional emission peaks remain unassigned at this stage, the PL results confirm that incorporation of Sn into Cs_3_Bi_2_Br_9_ significantly changes the host emission properties of the host material. The redshift of the transition energies is followed by an overall decrease of photoluminescence intensity, by more than two orders of magnitude for 23.5% Sn: Cs_3_Bi_2_Br_9_, indicating introduction of additional non-radiative recombination channels upon doping.

**Fig. 2:**
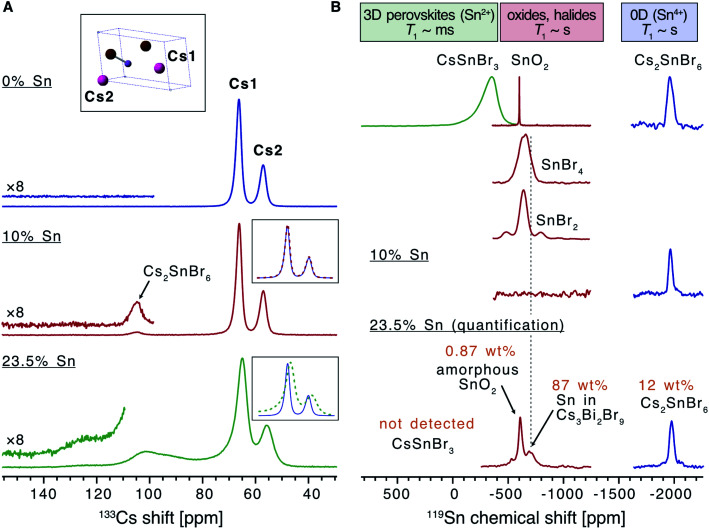
Solid-state NMR characterization of the materials. (A) ^133^Cs NMR spectra of the undoped Cs_3_Bi_2_Br_9_ and the materials doped with 10 and 23.5% Sn (only the region showing the isotropic resonances are shown). The inset shows the asymmetric unit cell of Cs_3_Bi_2_Br_9_ and the assignment of the cesium sites. (B) ^119^Sn NMR spectra of the 10% and 23.5% Sn-doped materials compared to a library of tin-containing compounds (tin(iv) oxide, tin(ii)/(iv) bromides, cesium halostannates(ii)/(iv)). Signals correspond to SnO_2_, Cs_2_SnBr_6_, and Sn within the Cs_3_Bi_2_Br_9_ structure are marked. The spectrum corresponding to the 23.5% Sn material is quantitative (see Table S2† for experimental details).

Solid state nuclear magnetic resonance (NMR) was used to analyse the effect of Sn doping on the local structure of the parent material (Fig. 2). There are two inequivalent Cs sites in the asymmetric unit of Cs_3_Bi_2_Br_9_ present in a 2 : 1 ratio, hence the ^133^Cs NMR spectrum of this compound can be readily assigned (Fig. 2A). While the effect of Sn doping is not detectable in the ^133^Cs signal in the 10% Sn material, the signals of Cs_3_Bi_2_Br_9_ in the 23.5% Sn sample are broadened compared with the undoped material, indicating that the atomic-level structure of the parent compound has changed after the introduction of Sn. The 10% sample contains a small amount of Cs_2_SnBr_6_, and the molar ratio of Cs_2_SnBr_6_ : Cs_3_Bi_2_Br_9_ is 4 : 96 based on the quantitative ^133^Cs spectrum. It has previously been found that the (practical) detection limit for iodide substituting bromide in a ternary metal compound by disorder-induced broadening of ^133^Cs NMR signals is around 2%.^58^ A similar limit may pertain here for substitutional Sn detection, although two factors suggest that ^133^Cs NMR could be less sensitive to doping in our case. Firstly, since the average Sn ionic radius is much more similar to that of Bi(iii) than the iodide radius is to the bromide radius, it is reasonable that Sn substitution would lead to less structural distortion. Secondly, substitution occurs in the second coordination shell of Cs, rather than the first as in the substitution of I with Br in the ternary metal halides described above. The broad convolution of ^133^Cs signals between 70 and 130 ppm in the 23.5% sample cannot be assigned unambiguously but is consistent with the presence of new Cs sites adjacent to the Sn dopants in the Sn-doped Cs_3_Bi_2_Br_9_ structure. A Cs environment consistent with the Cs_2_SnBr_6_ impurity is observed also in this sample.

In order to elucidate the speciation of tin in the doped materials, we turn to ^119^Sn MAS NMR. We have very recently overcome the challenges associated with the acquisition of tin NMR data on halostannates(ii)/(iv) and we apply those findings here.^40^ The chemical shift of ^119^Sn is a highly sensitive probe of the local Sn environment, with the oxides, halides, halostannates(ii) and halostannates(iv) resonating in distinct spectral ranges, as illustrated by the reference spectra of CsSnBr_3_, SnO_2_, Cs_2_SnBr_6_, SnBr_4_ and SnBr_2_ (Fig. 2B).^40^ The quantitative ^119^Sn solid-state MAS NMR spectrum of the 23.5% Sn material contains signals corresponding to multiple Sn-containing phases. The signal at −1976 ppm corresponds to Cs_2_SnBr_6_, and the signal at −608 ppm is assigned as SnO_2_, with the large line width of this signal suggesting that the SnO_2_ phase is characterised by high local disorder, *i.e.* either amorphous or nanocrystalline.^40,59^ Finally, there is a broad signal spanning the region between −480 and −800 ppm, which does not correspond to any other known phases. We therefore tentatively assign it to Sn sites within the Cs_3_Bi_2_Br_9_ structure whose presence is expected based on the ^133^Cs NMR data. If the two Sn valences were trapped and distinguishable by NMR, we would expect to see two signals corresponding to Sn(ii) and Sn(iv), approximately in the chemical shift range shown for CsPbBr_3_ and Cs_2_PbBr_6_, respectively. Instead, we observe a broad signal in the intermediate range, consistent with the valences fluctuating dynamically between the two limits, as elucidated by optical spectroscopy (*vide infra*). No metallic tin or CsSnBr_3_ were detected. In the sample with the highest Sn doping level, the amount of SnO_2_ and Cs_2_SnBr_6_ was quantified as 0.87 wt% and 12 wt% of the sample respectively (see ESI† for full details of this calculation). This sample was the highest doping level where secondary phases were not detected by XRD, yet the NMR results show that additional phases are indeed present. In the lower concentration Sn sample studied by NMR (10% Sn doping level), neither the broad signal corresponding to Sn in Cs_3_Bi_2_Br_9_, nor the SnO_2_ signal were observed. This result is consistent with the corresponding ^133^Cs spectrum, which was unchanged compared to pristine Cs_3_Bi_2_Br_9_, and indicates that the doping level is lower than the sensitivity limit of the technique. A signal corresponding to Cs_2_SnBr_6_ is observed, however, showing that some secondary Sn(iv) containing phase is present even at lower Sn doping levels in samples made by this method (Cs_2_SnBr_6_ : Cs_3_Bi_2_Br_9_ is 4 : 96 based on the quantitative ^133^Cs spectrum, as discussed above). Importantly, neither SnO_2_ nor Cs_2_SnBr_6_ have strong visible light absorption, and so the presence of these phases cannot explain the black colour of the samples. In addition, we have verified that the synthetic protocol is reproducible, with different batches yielding materials with essentially identical atomic-level structure (Fig. S10†). The amount of Sn that is doped into the Cs_3_Bi_2_Br_9_ lattice was calculated from the quantitative ^119^Sn NMR spectra. In the sample with an analytical Sn percentage of 23.5%, approximately 7% of the Bi sites are replaced with Sn, the remainder of the Sn is present in the secondary phases, Cs_2_SnBr_6_ and SnO_2_. We take this doping level of 7% to be the maximum possible Sn incorporation into Cs_3_Bi_2_Br_9_ under these synthesis conditions. Those samples with analytical Sn compositions lower than 23.5% but above 7% very likely also have lower Sn dopant concentrations and some secondary Sn containing phases. Since quantitative NMR is not available for all samples studied here, we continue to use analytical Sn concentrations to describe samples, but note that the true Sn dopant concentration is likely less than the analytical value. This is a good example of a case where powder XRD does not give a complete picture of the product identity, which may be a common situation in low temperature solution synthesised materials.

The presence of a new ^119^Sn signal, detectable in the higher Sn doped sample, and the broadening of the ^133^Cs signal are evidence that Sn has the capacity to incorporate into the structure of Cs_3_Bi_2_Br_9_. This result is consistent with the broadening of the Cs_3_Bi_2_Br_9_ XRD peaks with increasing Sn content and corroborates the incorporation of Sn into the Cs_3_Bi_2_Br_9_ structure. Variable-temperature NMR studies are expected to shed more light on the dynamics of the valence fluctuations.

**Fig. 3:**
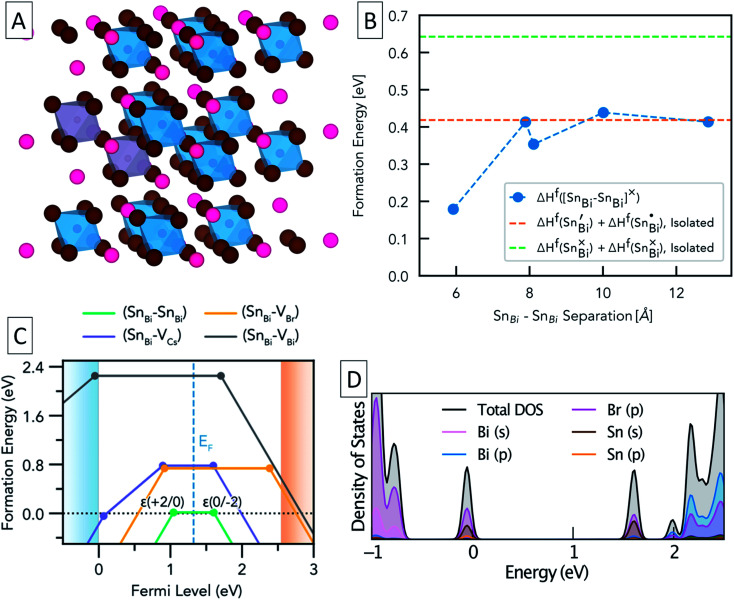
(A) Structure of Cs_3_Bi_2_Br_9_ showing two substitutional Sn defects (Sn_Bi_–Sn_Bi_) on nearest neighbour sites (grey octahedra). (B) Formation energy of (Sn_Bi_–Sn_Bi_)^×^ on different relative positions, compared with the energies of two isolated Sn substitutional defects (horizontal dashed lines). The nearest-neighbour double substitution complex is the most energetically favourable. (C) Defect formation energy diagram for the (Sn_Bi_–V_X_); X = Cs, Bi, Br; and (Sn_Bi_–Sn_Bi_) complexes in Cs_3_Bi_2_Br_9_, under Sn-rich conditions. Valence band in light blue, conduction band in light orange. Vertical dashed steel-blue line indicates position of the self-consistent Fermi level, and filled circles indicate thermodynamic charge transition energy levels. (D) Orbital-projected electronic density of states for the (Sn_Bi_˙–Sn_Bi_^/^) double-substitution complex in Cs_3_Bi_2_Br_9_ for an atomic Sn_Bi_ concentration of 12.5%. Position of zero occupancy set to 0 eV.

**Fig. 4:**
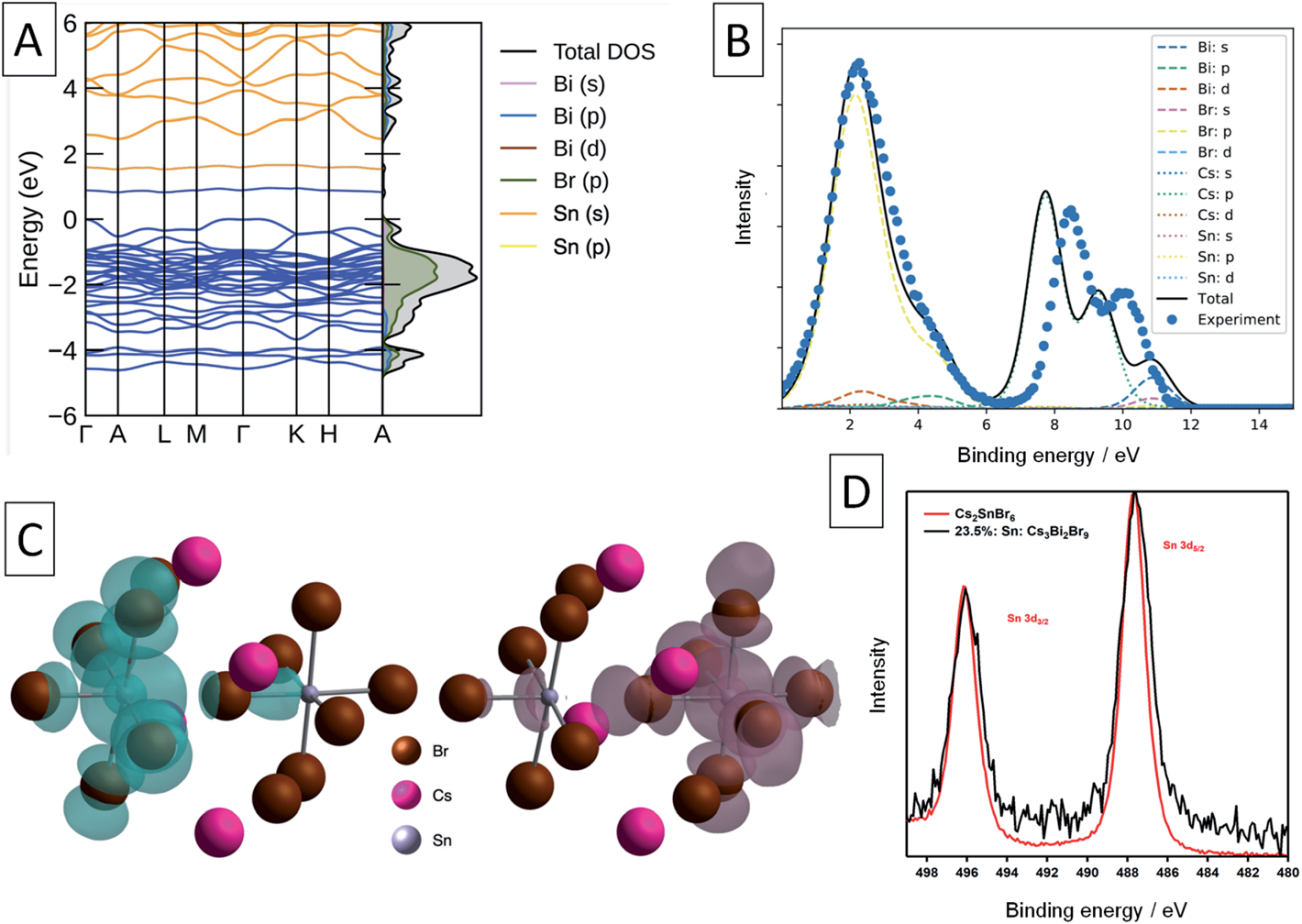
Calculated and experimental electronic structure of Sn-doped Cs_3_Bi_2_Br_9_. (A) Calculated electronic band structure of Sn-doped Cs_3_Bi_2_Br_9_, with the corresponding electronic density of states plotted alongside vertically. Filled states in blue, empty states in orange. The valence band maximum (VBM) is set to 0 eV. (B) Experimental XPS valence band spectrum (blue points) and simulated spectrum from the calculated electronic structure. The experimental spectrum has been shifted 1.40 eV to lower binding energy to align the valence band maximum. (C) Charge density isosurfaces of the occupied (left) and unoccupied (right) intra band gap states. Isovalue set to 0.01 e Å^−3^. (D) XPS Sn 3d core line comparison between Cs_2_SnBr_6_ and Sn-doped Cs_3_Bi_2_Br_9_.

To further study the nature of Sn dopants within the Cs_3_Bi_2_Br_9_ structure, defect calculations were used to assess the most stable defects present in the doped material. Initially, the defect formation energy diagram was calculated for an isolated Sn_Bi_ defect in the neutral and singly-charged states, with the result shown in Fig. S3.† As expected, the single isolated Sn_Bi_ substitutional defect is found to exhibit negative-U behaviour, with +1, Sn(iv), and −1, Sn(ii), the only stable charge states for all Fermi level positions in the band gap. Both the negative-U behaviour of isolated Sn_Bi_ and the high levels of doping in the experimental samples suggested that atomic-scale clustering of Sn_Bi_ substitutions to form double-substitutional complexes, (Sn_Bi_–Sn_Bi_), was likely. To investigate this, the formation energy for two Sn_Bi_ substitutions was calculated for all possible separations (*i.e.* B ion crystal sites) within the size constraints of the 112-atom supercell (allowing Sn_Bi_ separations up to 16 Å to be computed), with the results shown in Fig. 3. These calculations indicate a clear preference for Sn_Bi_ to pair in nearest-neighbour arrangements, forming (Sn_Bi_–Sn_Bi_) double-substitution complexes as shown in Fig. 3A. A small second energy minimum appears at ∼8.1 Å separation, corresponding to the next-nearest neighbour substitution site within the same BX_6_ octahedral layer, whereas the ∼7.9 Å separation corresponds to Sn_Bi_ substitutions located in neighbouring BX_6_ layers. The lower energy of the ∼8.1 Å separation indicates more favourable defect–defect interactions when mediated through the quasi-2D BX_6_ framework. Fig. 3C shows the formation energies of the lowest-energy arrangement for each possible Sn_Bi_–vacancy complex, in addition to the (Sn_Bi_–Sn_Bi_) double-substitution complex, at the Sn-rich limit. The (Sn_Bi_–Sn_Bi_) double-substitution complex is predicted to be by far the most thermodynamically-favourable defect species of those investigated, lying ∼0.8 eV lower in energy than the (Sn_Bi_–V_Br_) and (Sn_Bi_–V_Cs_) defects at the self-consistent Fermi level. In the Sn_Bi_ double-substitution complex, the Sn atoms are found to disproportionate, forming the mixed-valence complex (Sn_Bi_˙ − Sn_Bi_^/^). In this arrangement, the Sn_Bi_ substitutions introduce two distinct electronic states within the bandgap (Fig. 3d and 4a, c), arising from Sn s–Br p hybridisation (as well as some Sn p character for the occupied, lower energy state).

The formation energy of (Sn_Bi_˙–Sn_Bi_^/^) was calculated using two defect supercell sizes, corresponding to atomic Sn concentrations of 12.5% and 25% respectively (see computational methodology). The formation energy of (Sn_Bi_˙–Sn_Bi_^/^) was found to be 72 meV higher for the 25 at% Sn material compared with the 12.5% Sn material, suggesting that Sn incorporation becomes less favourable at higher concentrations, in good agreement with experimental observations of decreased Sn incorporation and secondary phase formation at higher nominal Sn concentrations.

In the (Sn_Bi_–Sn_Bi_) double-substitution complex, the formal oxidation states of the Sn atoms can be assigned as IV and II respectively. For comparison, we have calculated the oxidation state assignments, according to Bader charge density partitioning, for this defect species as well as several similar tin-bromide materials, with the results provided in Table 1.

**Table 1:** Formal oxidation state and calculated charge state from Bader analysis for Sn species in Sn-doped Cs_3_Bi_2_Br_9_ and several representative tin-bromide materials

Material	Species (formal oxidation state)	Bader charge state
Cs_3_(Sn_0.125_Bi_0.875_)_2_Br_9_	Sn(iv)	1.77
Cs_3_(Sn_0.125_Bi_0.875_)_2_Br_9_	Sn(ii)	1.18
Cs_3_(Sn_0.25_Bi_0.75_)_2_Br_9_	Sn(iv)	1.78
Cs_3_(Sn_0.25_Bi_0.75_)_2_Br_9_	Sn(ii)	1.18
CsSnBr_3_	Sn(ii)	1.19
CsSn_2_Br_5_	Sn(ii)	1.22
SnBr_2_	Sn(ii)	1.24
SnBr_4_	Sn(iv)	1.71
Cs_2_SnBr_6_	Sn(iv)	1.78

A Bader charge state of ∼1.2 is consistent with that of Sn in other formal Sn(ii) compounds (such as SnO).^16^ However, the observed Bader charges of ∼1.8 in the above formal Sn(iv)–Br compounds is significantly lower than that observed for Sn(iv) in SnO_2_ (∼2.4),^60^ demonstrating the strong degree of covalency and charge delocalisation within the Sn(iv)–Br motif (see Fig. 4c).

XPS was used in an attempt to determine the oxidation state of Sn in these compounds. The Sn 3d XPS spectra were compared to those for a similar Sn(iv) compound, the vacancy-ordered double perovskite Cs_2_SnBr_6_, synthesised in our previous work and are shown in Fig. 4d.^24^ The full width at half-maximum (FWHM) of the Sn 3d_5/2_ core line in Sn: Cs_3_Bi_2_Br_9_ averages to 1.74 eV across the samples synthesised, which is larger by 0.38 eV than the value of 1.36 eV obtained for Cs_2_SnBr_6_. In contrast, the Cs 3d peak FWHM of 1.59 eV in mixed-valent Sn: Cs_3_Bi_2_Br_9_ remains virtually unchanged from that of Cs_2_SnBr_6_ at 1.60 eV. Whilst the peaks corresponding to the different Sn valencies are not resolved due to the small chemical shift between Sn(ii) and Sn(iv),^61^ this broadening can be attributed to the appearance of the lower binding energy peak corresponding to the divalent tin.

Thus, our experimental and computational results establish that divalent tin incorporated into the solution phase synthesis of Cs_3_Bi_2_Br_9_ results in a mixed-valent compound with a characteristic double-substitution defect motif in which Sn ions of different valency occupy nearest neighbour sites. These nearest-neighbour B cation sites are found within the plane of the 2D bismuth halide corner-sharing octahedral network, meaning that each (Sn_Bi_–Sn_Bi_) double substitution complex exists within these planes.

Turning to a discussion of the electronic structure of these materials, we calculate that undoped Cs_3_Bi_2_Br_9_ has a fundamental indirect electronic gap of 2.55 eV, and a direct optical gap of 2.66 eV, in excellent agreement with our experimental measurement of the band gap at 2.58 eV, and previous measurements discussed earlier.^54^ The calculated electronic band structure of Sn doped Cs_3_Bi_2_Br_9_ is shown in Fig. 4. As stated above, the Sn atoms in the double-substitution complex are found to disproportionate, forming the mixed-valence complex with neighbouring Sn(ii) and Sn(iv) ions which has overall neutral charge compared with the substituted Bi(iii) ions: we denote this defect complex as (Sn_Bi_˙–Sn_Bi_^/^) using Kroger–Vink notation. This produces a fully-occupied state (associated with the Sn_Bi_^/^site) and fully-unoccupied electronic state (associated with the Sn_Bi_˙ site) within the Cs_3_Bi_2_Br_9_ bandgap (Fig. 3d and 4a, c).

The intra-gap charge transition levels of (Sn_Bi_–Sn_Bi_), *ε*(+2/0) and *ε*(0/−2), are located at 1.04 and 1.60 eV above the VBM, respectively. Both of these defect levels are ‘negative-U’ states – *i.e.* the defect can trap two holes/electrons with the second bound more strongly than the first. As such, the singly-charged states, corresponding to (Sn_Bi_˙–Sn_Bi_^×^) and (Sn_Bi_^×^–Sn_Bi_^/^), are predicted to be thermodynamically unstable at all Fermi level positions – at *ε*(+2/0) (Sn_Bi_˙–Sn_Bi_^×^) is 34 meV higher in energy than the neutral or +2 charge state while at *ε*(0/−2) (Sn_Bi_^×^ −Sn_Bi_^/^) is 16 meV higher in energy than the neutral or −2 charge state (see Section B in the ESI† for more on this).

From the predicted charge-dependent defect formation energies in Fig. 3C, the self-consistent Fermi level is calculated to lie at 1.32 eV above the VBM. Due to the dominance of the (Sn_Bi_–Sn_Bi_) defect species, the self-consistent Fermi level is entirely determined from the positions of the charge transition levels of this double substitution complex – lying exactly at their midpoint, just above the intrinsic midgap position (of 1.275 eV above the VBM). At this Fermi level, essentially all defect species are present only in the neutral charge state. The predicted concentrations are provided in Table 2.

**Table 2:** Calculated equilibrium concentrations of (Sn_Bi_–V_X_); X = Cs, Bi, Br; and (Sn_Bi_–Sn_Bi_) complexes in Cs_3_Bi_2_Br_9_ at the self-consistent Fermi level (*E*_F,self-consistent_ = 1.32 eV above VBM), under Sn-rich conditions at room temperature (*T* = 300 K)

Defect species	(Sn_Bi_˙–Sn_Bi_^/^)	(Sn_Bi_^/^–V_Br_˙)^×^	(Sn_Bi_˙^–^V_Cs_^/^)^×^	(Sn_Bi_˙^–^V_Bi_^//^)^×^
Concentration (cm^−3^) at *E*_F,self-consistent_	1.0 × 10^21^	2.3 × 10^11^	4.6 × 10^9^	0

We compare the calculated electronic structure with the experimental XPS valence band spectrum in Fig. 4. The calculated density of states was converted to a simulated XPS spectrum using Galore,^62^ with the valence band maximum set at a binding energy of 0 eV. To align the spectra, a −1.4 eV shift was applied to the experimental binding energies shown in Fig. 4 in order to achieve the best match with the simulated spectrum. Since experimentally, the XPS binding energy scale is referenced to the Fermi level, this 1.4 eV shift is a measure of the energy difference between the VBM and the Fermi level, and matches closely with that predicted from our defect calculations (1.32 eV). The calculated spectrum matches well with the experimental spectrum in the 0–6 eV binding energy region, with some divergence in the binding energy of the Cs 5p shallow core lines.

## Optical properties

In light of the results obtained thus far, we turn to a brief discussion of the Robin–Day classification for mixed valency compounds. Class I encompasses materials where the valences are trapped, *i.e.*, localised on a single site.^38^ There are distinct sites with different specific valences in the complex that cannot easily interconvert, resulting in no electrical conductivity, and an absorption spectrum which is the simple sum of the constituent ions. A typical example of a Class I compound is Pb_3_O_4_. At the other extreme lies Class III, where mixed valence is not distinguishable by spectroscopic methods, as the valence is completely delocalised. Each site exhibits an intermediate oxidation state, which can be half-integer in value. This class is possible when the ligand environment is similar or identical for each of the two metal sites in the complex. The resulting materials do not behave as metals or semiconductors, nor do they exhibit “single-ion” properties, such as electronic, vibrational, or Mössbauer spectra characterising the metal in either of its oxidation states. They do usually exhibit strong optical absorption caused by electron hopping between sites. The Creutz–Taube complex and sodium tungsten bronzes are typical examples of a Class III compound.

Lastly, Class II compounds are characterised by sites occupied by ions differing in valence which are similar, yet still distinguishable.^38^ There is some localisation of distinct valences, albeit with a low activation energy for their interconversion. Some thermal activation is required to induce electron transfer from one site to another, meaning that Class II compounds are semiconductors. The electron delocalisation is small, and single-ion properties persist in these mixed valence systems, often with very little perturbation, resulting in a practically unchanged vibrational frequency of the constituent ions. Like Class II compounds, Class II materials exhibit an intense intervalence charge transfer band, and therefore a broad and intense absorption in the infrared or visible part of the spectrum, as well as magnetic exchange coupling at low temperatures. Vanadium oxides and the Fe(ii)/Fe(iii) cyanide complex, Prussian blue, are well known examples of Class II compounds.

Our calculations show that the disproportionated defect consisting of Sn(ii) and Sn(iv) ions is the ground state of this material. Together with the presence of a strong visible light absorption band, we propose that Sn: Cs_3_Bi_2_Br_9_ is a Robin–Day Class II material. It is also possible that the material undergoes a Verwey transition to a Class III compound below room temperature, as occurs, for example, in Fe_3_O_4_;^36,63^ elucidation of this point is beyond the scope of this current work. Of note is that Sn: Cs_3_Bi_2_Br_9_ displays some important differences compared to the classical Robin–Day compounds previously investigated. In particular, Cs_2_Sn_*x*_Sb_1−*x*_Cl_6_, which contains Sb(iii) and Sb(v) ions, provides an interesting comparison. The energy of the IVCT absorption maximum is determined to increase with increasing Sb concentration for Sb: Cs_2_Sn_2_Cl_6_, and many previously studied mixed valence systems mimic this trend (Fig. S8†). In this regard, Sn: Cs_3_Bi_2_Br_9_ is highly unusual as it displays the opposite trend in its optical properties.

**Fig. 5:**
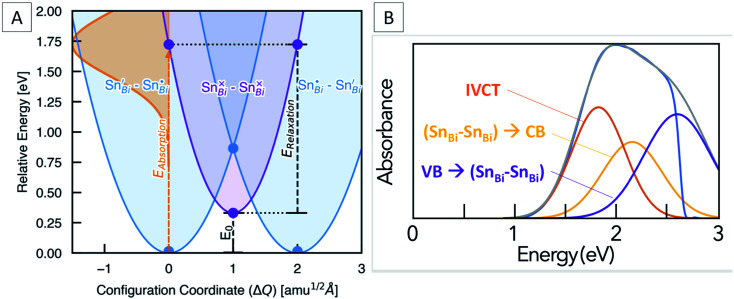
(A) Configuration coordinate diagram for the (Sn_Bi_˙–Sn_Bi_^/^) to (Sn_Bi_^×^–Sn_Bi_^×^) IVCT transition, showing the calculated optical excitation (*E*_absorption_), vibrational relaxation (*E*_relaxation_) and thermodynamic transition (*E*_0_) energies. The solid lines are harmonic fits to the DFT energies, represented by filled circles. *X*-Axis given in units of mass-weighted displacement. (B) Difference absorption spectrum of Sn doped and undoped Cs_3_Bi_2_Br_9_, fitted with three components corresponding to the IVCT absorption and two ‘band to band gap state’ transitions at energies calculated from DFT.

Marcus–Hush theory was developed to understand rates of electron transfer in redox processes, but has been successfully adapted to model electron transfer in mixed valence systems.^64,65^ For our system, the ground states (Sn_Bi_˙–Sn_Bi_^/^) and (Sn_Bi_^/^–Sn_Bi_˙) are of equal energy, and are shown as the blue potential energy parabolas in Fig. 5A. Transfer of one electron from Sn_Bi_^/^to Sn_Bi_˙ generates the excited state (Sn_Bi_^×^–Sn_Bi_^×^), consisting of two Sn(iii) ions, this is represented by the purple parabola in Fig. 5A. According to the two-level Hush model for intervalence charge transfer, two mechanisms of charge transfer, optical and thermal, occur. The former is the direct Frank–Condon electron transition from the ground state energy surface to the final state energy surface. This gives rise to intervalence charge transfer observed in the absorption spectra of mixed-valence materials. The second mechanism is a thermally induced intervalence charge transfer, which occurs by moving the pair along the ground state energy surface by an amount known as the reorganisation energy, which can be regarded as lowering of the difference between the mean metal–ligand distances in adjacent donor and acceptor polyhedra due to thermal vibrations. For weakly-coupled symmetrical mixed valence systems, the energy barrier for thermal electron transfer, *E*_a_, is directly related to the optical process through the maximum energy of the IVCT band, *ν*_max_, as follows:

1 *v*_max_ = 4*E*_a_

For symmetric one-electron transfers, where the initial and final states are identical, it is predicted that the Franck–Condon maximum of the optical transition occurs at four times the activation energy of the thermal transfer. For most Class II systems, and particularly asymmetric and many-electron transfer reactions, the energy difference between the initial and final states and therefore the equilibrium energy, *E*_0_, is not zero, and so:

2 *v*_max_ = 4*E*_a_ − *E*_0_

In our case, *E*_0_ is the difference in energy between the (Sn_Bi_˙–Sn_Bi_^/^) ground state and the (Sn_Bi_^×^–Sn_Bi_^×^) excited state (Fig. 5A). The optical absorption maxima we observe range from 2.08 eV at low Sn concentration to 1.90 eV at the highest Sn concentration. This compares well with our calculations, which places the energy of the optical electronic transition from (Sn_Bi_˙–Sn_Bi_^/^) to (Sn_Bi_^×^–Sn_Bi_^×^) at 1.72 eV for a concentration of 12.5 at% Sn in Cs_3_Bi_2_Br_9_, and 1.68 eV for the 25 at% Sn case (Fig. 5A). The calculations thus reproduce an unusual feature of the optical absorption trend, that of decreasing absorption energy with increasing dopant concentration; this is opposite to the trend observed in many previously studied mixed valence systems (Fig. S8†). The origin of decreased IVCT absorption energy with greater Sn doping content is primarily a greater spin-splitting of the partially-occupied Sn_Bi_^×^ states near the CBM (0.67 eV for the 12.5 at% Sn material, compared to 0.70 eV for the 25 at% Sn material – see Fig. 4), due to greater presence of the optically-excited high-spin (Sn_Bi_^×^–Sn_Bi_^×^) species. The higher concentration of (Sn_Bi_^×^–Sn_Bi_^×^) species in the highly-doped optically-excited material, and thus reduced spatial separation with nearby excited complexes, facilitates enhanced spin–spin interactions and thus a slight stabilisation of the excited state.

We also calculate the relaxation energy of the optically-excited (Sn_Bi_^×^–Sn_Bi_^×^) state as 1.39 eV. Subtracting this from the calculated optical absorption energy gives the thermodynamic transition energy, *E*_0_, from (Sn_Bi_˙–Sn_Bi_^/^) to (Sn_Bi_^×^–Sn_Bi_^×^) as 0.33 eV, for a 112-atom supercell corresponding to 12.5 at% Sn doping.

From Marcus-Hush theory, the optical band halfwidth of the IVCT band, Δ*ν*_1/2_, can be related to the equilibrium energy, *E*_0_. In the high-temperature limit, where the optical absorption band becomes nearly Gaussian, the equilibrium energy is related to this value by:^64,65^3
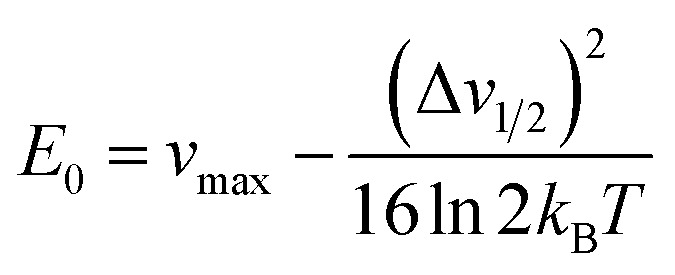
where *k*_B_ is the Boltzmann constant and *T* the temperature. Attempts to measure this bandwidth experimentally is hindered by the likely presence of other absorption features, as discussed below. However, by taking the calculated value of *E*_0_ for 12.5% Sn doping (0.33 eV), and the experimentally determined *ν*_max_ for the 12% Sn sample (1.92 eV), the calculated halfwidth, Δ*v*_1/2_, using eqn (3) is 0.68 eV. A Gaussian function with these parameters should then represent the IVCT absorption from (Sn_Bi_˙–Sn_Bi_^/^) to (Sn_Bi_^×^–Sn_Bi_^×^). Fig. 5B shows a difference spectrum between 23.5% Sn: Cs_3_Bi_2_Br_9_ and undoped Cs_3_Bi_2_Br_9_; this difference represents the additional absorption conferred by Sn doping compared to the host material. The calculated Gaussian IVCT absorption is an excellent fit to the low energy absorption edge of the difference spectrum, however, a significant amount of higher energy optical absorption is unaccounted for by IVCT. In attempts to investigate the discrepancy, we calculate the energies of optical transitions from the VBM to the vacant band gap state (corresponding to Sn_Bi_˙), and from the filled band gap state (corresponding to Sn_Bi_^/^) to the CBM. For the electronic transitions from (Sn_Bi_˙–Sn_Bi_^/^) to (Sn_Bi_^×^–Sn_Bi_^/^) + h_VBM_^+^ and from (Sn_Bi_˙–Sn_Bi_^/^) to (Sn_Bi_˙–Sn_Bi_^×^) + e_CBM_^−^, the absorption energies were calculated as 2.60 eV and 2.15 eV, respectively, for a concentration of 12.5% Sn in Cs_3_Bi_2_Br_9_, and 2.50 eV and 2.30 eV for the 25% Sn case. Fitting of one Gaussian function for each of these transitions, as shown in Fig. 5B, leads to a good fit of the difference spectrum in the region below the Cs_3_Bi_2_Br_9_ band edge, and the computed transitions may well be responsible for the portion of the absorption spectrum unaccounted for by the IVCT absorption.

## Stability

The stability of the Sn-doped Cs_3_Bi_2_Br_9_ materials is of interest, as Sn(ii) halides and halostannates(II) are typically prone to oxidation in air. Samples were stored in air in glass sample vials for extended periods (>12 months) without any noticeable changes to their appearance. XRD, XPS and UV-visible spectra were unchanged within error after 12 months of storage. However, mechanical grinding of the samples could lead to a rapid loss of the black colour and reversion to yellow associated with undoped Cs_3_Bi_2_Br_9_. ^133^Cs NMR measurements carried out on the powder after grinding revealed almost no change in signal compared with the freshly made material, suggesting that the Sn remains present within the Cs_3_Bi_2_Br_9_ phase, but no longer causes the strong visible light absorption, consistent with oxidation of the Sn(ii) upon grinding. Attempts to deposit films by solution phase methods were hindered, as dissolution of Sn-doped Cs_3_Bi_2_Br_9_ samples in conventional solvents (DMF, DMSO) caused rapid loss of the black colour, and films deposited from these solutions were only slightly darker than those of undoped Cs_3_Bi_2_Br_9_ (Fig. S12, S13, ESI†). We interpret this as the oxidation of the Sn(ii) in presence of solvents and also at high annealing temperatures (∼200 °C).^66^

Several properties of the doped material likely contribute to this unusual stability of the Sn(ii) species. Firstly, it should be noted that low-dimensional structures have previously been implemented as a means of enhancing material and device stability in conventional lead halide,^67^ as well as highly oxidation sensitive divalent tin-based perovskites systems.^68^ It is thought that slicing the full three-dimensional (3D) structure into layers delays oxygen and water diffusion within the lattice, thus enhancing stability. Secondly, the large electron-phonon coupling inherent to this class of layered A_3_B_2_X_9_ perovskite,^19^ reflected in the large vibrational relaxation energy for the IVCT of 1.39 eV, results in substantial oxidation-state dependence of the Sn_Bi_ coordination environment (>0.5 Å difference in Sn(ii)–Br/Sn(iv)–Br bond lengths). This, coupled with the strong hybridisation of Sn 5s & 5p with Br 4p and high energy VBM due to Bi s–Br p interactions^69^ (Fig. 3D, 4C, S6†), splits the Sn(ii)/Sn(iv) levels by over 1.5 eV and stabilises the Sn(ii) ‘lone-pair’ state within the lower-energy region of the bandgap. Lastly, the stability of the Sn-doped material may also be linked to the surface behaviour, including the ability of Cs_3_Bi_2_Br_9_ to self-passivate its surface with a layer of BiOBr,^70^ and the further inhibition of an inward oxidation cascade due to the dilute Sn(ii) concentration. Each of these three factors may contribute to the stability of Sn(ii) seen in these compounds. While the surface passivation and 2D layering that work by physically preventing contact between Sn(ii) and an oxidising species are understood from research on similar materials systems, the stability conferred on the Sn(ii) state by the electronic structure of the material reported here may form the basis for an important principle for design of air stable ternary metal halides in general.

## Discussion

Ternary metal halides were among the first mixed-valence compounds studied systematically, in classic work by Robin, Day and contemporaries. The central motif of this early work was replacement of a M(IV) ion in a cubic A_2_BX_6_ structure with a mixture of M′(III) and M′′(V). If M′ and M′′ are to be the same element, a convenient choice is the post transition metal (PTM) antimony, which can readily adopt its ‘lone pair’ oxidation state Sb(III) and its group oxidation state Sb(V). This led to compounds such as Cs_2_Sn_(1−*x*)_Sb_*x*_X_6_ which revealed fundamental features of mixed valence chemistry, the study of which continues today.^37,39,71^

The phenomenon of valency disproportionation in two electron difference mixed valence systems may appear to be surprising, as it necessitates the pairing of electrons on the same site, and consequently involves large electron repulsions. The “negative *U* effect” is therefore the outweighing of the repulsion, *U*, of the two electrons occupying the same orbital by the gain in lattice Coulomb energy, as well as the gain in elastic energy associated with strong electron phonon coupling. Hence, valency disproportionation at the expense of *U* tends to occur for electrons with small free-atom *U*, such as the 5s electrons in the PTMs utilised here. Crucially, in order to overcome the large values of *U*, large vibronic coupling constants are necessary.^72,73^ Strong electron-phonon coupling has recently been reported for a group of related A_3_B_2_X_9_ compounds,^19,74^ and this may be a vital feature of the 2D A_3_B_2_X_9_ structure that permits the mixed valence doping we observe here.

Perhaps surprisingly, given the highly influential nature of the early mixed-valence work by Robin, Day and co-workers, the strategy of replacing a metal ion in a ternary halide of oxidation state N with a PTM in oxidation states (N−1) and (N+1) was not extended to other PTMs, until recent work on CsTlCl_3_ and CsInCl_3_, both mixed-valence M′(i)/M′′(iii) compounds.^32,75,76^ In the current work, we have extended this strategy further to partial replacement of Bi(iii) with the group 14 metal Sn, which shows two oxidation states of +2 and +4, with the latter usually being more stable .

Similar approaches have been used to improve visible light absorption in lead free halide semiconductors, however. Roy *et al.* incorporated Pb into Cs_3_Bi_2_Br_9_, and found that the predominant Pb species present was Pb(ii), forming the defect complex Pb_Bi_ + V_Br_,^77^ rather than a mixed oxidation state complex that we observe with Sn doping. Correspondingly, the Pb doped Cs_3_Bi_2_Br_9_ showed a minimal band gap reduction to 2.23 eV (from 2.6 eV in Cs_3_Bi_2_Br_9_). Bi(iii) doping into (CH_3_NH_3_)PbBr_3_ was initially thought to reduce the band gap, although subsequent careful measurements showed this effect was due to localised intra-bandgap states.^78^ On the other hand, Ju *et al.* doped (CH_3_NH_3_)_3_Sb_2_I_9_ with Sn, and found a significant redshift in the absorption edge.^79^ The host compound forms in the 0D dimer structure, and Sn(ii) was identified as the dopant responsible for the optical change. However, it appears that DFT calculations used to support this conclusion were only carried out with one Sn ion in the supercell, which would preclude the identification of mixed valence Sn ground state as we have found here. Lindquist *et al.* found that Sn doping of the double perovskite Cs_2_BiAgBr_6_ led to redshift of the band gap.^27^ The authors conclude that both Sn(ii) and Sn(iv) are present, but do not attribute the optical changes to any form of IVCT. In another study, Ghosh *et al.* synthesised the mixed valence perovskite (CH_3_NH_3_)_2_Au_2_I_6_ and showed a significant redshift in the absorption spectra, which they linked to IVCT.^29,80^ This is similar to Cs_2_Au_2_X_6_ series of compounds reported some years ago.^34^ Lastly, very recently Benin *et al.* reported a series of mixed valence Sb compounds with formula Rb_23_Bi_*x*_Sb_9–*x*_Cl_54_ (0 ≤ *x* ≤ 7), which can be described as Bi doped Rb_2_SbCl_6_, although the structure is significantly more complex than the parent cubic phase.^81^ The Bi doping led to lower resistivity than corresponding mixed valence A_2_BX_6_ compounds.

The examples cited above show that in a few cases, IVCT has been explicitly used with the aim of increasing visible light absorption or charge transport in optoelectronic materials, and in other cases, it may play a role that was not explicitly elucidated. While Ju *et al.* found that Sn doping of (CH_3_NH_3_)_3_Sb_2_I_9_ led to an increase in electron mobility, it has not so far been established whether the visible light absorption that arises from IVCT is useful for energy harvesting. While we cannot address this point conclusively with the results here, we show that in addition to IVCT, the filled and empty states created within the band gap by mixed valence Sn doping lead to transitions more characteristic of intermediate band (IB) solar cells:^82,83^*i.e.*, a valence band to intermediate band transition, and an intermediate band to conduction band transition. Although our initial attempts at translating these Sn-doped Cs_3_Bi_2_Br_9_ powders to corresponding thin films compatible with solar cells were unsuccessful, possibly due to the rapid oxidation of Sn(ii) in solution and also during film annealing, alternative strategies for thin film deposition will hopefully be reported in future to bridge this gap. Further work will also be required to explain the nature of the emission features observed at low temperature.

## Conclusion

In conclusion, we have synthesised and characterised a novel mixed valency ion doped vacancy-ordered triple perovskite, Sn:Cs_3_Bi_2_Br_9_, which expands the structural and compositional space of Class II Robin–Day compounds. The material features broadband absorption which, through supporting calculations, we ascribe to an inter-valence charge transfer transition. We also identify possible intermediate band transitions involving the band edges and Sn based states within the band gap. Powder samples are stable in air for long periods, which may be due to surface effects alongside the enhanced stability of the Sn(ii) ion conferred by the stabilising electronic and elastic interactions associated with the double substitution. Uniquely among the previously studied mixed valence metal halides, we find the mixed valence dopants prefer to associate in an electrically neutral defect complex, and this leads to differences in the evolution of the IVCT band compared with classical materials such as Cs_2_Sn_1−*x*_Sb_*x*_Cl_6_. While we speculate that the IVCT absorption itself may not produce extractable charge carriers, the associated intermediate band transitions may be able to do so, and this could be a topic for further study to assess the photovoltaic potential of such materials.

## Data availability

All calculation data and analyses are provided in an online repository at https://doi.org/10.5281/zenodo.5507962.

## Author contributions

The author contributions are defined below according to the CRediT contributor roles taxonomy. Conceptualization: Chantalle J. Krajewska, Seán R. Kavanagh, Robert G. Palgrave. Investigation and methodology: Chantalle J. Krajewska, Seán R. Kavanagh, Lina Zhang, Dominik J. Kubicki, Krishanu Dey, Krzysztof Gałkowski. Validation: Lina Zhang. Supervision, resources and funding acquisition: Clare P. Grey, Samuel D. Stranks, Aron Walsh, David O. Scanlon, Robert G. Palgrave. Software: Seán R. Kavanagh, Aron Walsh, David O. Scanlon. Writing – original draft: Chantalle J. Krajewska, Seán R. Kavanagh. Writing – review & editing: All authors. Project administration: Robert G. Palgrave.

## Conflicts of interest

There are no conflicts of interest.

## Supplementary Material

SC-012-D1SC03775G-s001
