# Supplementary material for: Enhanced visible light absorption in layered Cs3Bi2Br9 through mixed-valence Sn(ii)/Sn(iv) doping
Source: Chem Sci. 2021 Oct 5;12(44):14686–99. doi: 10.1039/d1sc03775g (PMC8597838; doi:10.1039/d1sc03775g)
Supplement: SC-012-D1SC03775G-s001 [file SC-012-D1SC03775G-s001.pdf]

Electronic Supplementary Material (ESI) for Chemical Science.
This journal is © The Royal Society of Chemistry 2021

Supporting Information: Enhanced visible light absorption in layered $Cs_3Bi_2Br_9$ through mixed-valence Sn(II) / Sn(IV) doping

Chantalle J. Krajewska,[a] Seán R. Kavanagh,[a,b,c] Lina Zhang,[a] Dominik J. Kubicki,[d,j] Krishanu Dey,[d] Krzysztof Gałkowski,[d,e,f] Clare P. Grey,[j] Samuel D. Stranks,[d,g] Aron Walsh,[c,h] David O. Scanlon,[a,b,i] Robert G. Palgrave[a]*

a) Department of Chemistry, University College London, 20 Gordon Street, London, WC1H 0AJ

b) Thomas Young Centre, University College London, Gower Street, London WC1E 6BT, United Kingdom

c) Department of Materials, Imperial College London, Exhibition Road, London SW7 2AZ, UK

d) Cavendish Laboratory, University of Cambridge, JJ Thomson Avenue, Cambridge CB3 0HE

e) Institute of Physics, Faculty of Physics,Astronomy and Informatics, Nicolaus Copernicus University, 87-100 Toruń, Poland

f) Department of Experimental Physics, Faculty of Fundamental Problems of Technology, Wroclaw University of Science and Technology, 50-370 Wroclaw, Poland

g) Department of Chemical Engineering & Biotechnology, University of Cambridge, Philippa Fawcett Drive, Cambridge CB3 0AS

h) Department of Materials Science and Engineering, Yonsei University, Seoul 03722, Korea

i) Diamond Light Source Ltd., Diamond House, Harwell Science and Innovation Campus, Didcot, Oxfordshire OX11 0DE, United Kingdom

j) Department of Chemistry, University of Cambridge, Lensfield Road, Cambridge CB2 1EW, UK

*corresponding author email: r.palgrave@ucl.ac.uk

Computational Details

The computational methods are described in the main manuscript. Below is additional information.

For defect calculations involving Sn_B – V_X (X = Cs, Bi, Br), a 56-atom supercell was used, whilst for (Sn_{Bi} – Sn_{Bi}) and isolated Sn_{Bi} defects, both a 56-atom and a 112-atom supercell were employed in order to investigate concentration effects, produced from 2 x 2 x 1 and 2 x 2 x 2 cubic expansions of the hexagonal $Cs_3Bi_2Br_9$ unit cell. The same plane-wave energy cutoff (350 eV) was employed as for bulk electronic structure calculations, with a 2 x 2 x 1 Γ-centred Monkhorst-Pack **k**-point mesh (equivalent to a **k**-point density of 0.31 $Å^{-1}$).

For the 112-atom 2x2x2 supercell, 'rattling' the non-defective structure by applying random atomic displacements following a normal distribution (σ = 0.25 Å), prior to geometry relaxation, resulted in octahedral tilting and a final energy 0.256 eV (2.3 meV/atom) lower than the unperturbed structure. As such, 'rattling' was applied to each defective 112-atom supercell and compared to the 'rattled' non-defective supercell when calculating these defect formation energies, to ensure the global minimum on the structural potential energy surface was obtained.

To account for spurious finite-size supercell effects, a modified version of the Kumagai-Oba anisotropic charge correction scheme (eFNV) was implemented, with a 2/3 scaling of the point-charge correction energy, which has been shown to produce accurate results for defect formation energies.[1-3] If necessary, Moss-Burstein type band filling corrections were also applied. For the calculation of optical transitions involving a change in defect supercell charge (*i.e.,* electronic transitions from ($Sn_{Bi}^{\bullet}$ – $Sn_{Bi}^{/}$) to ($Sn_{Bi}^{\times}$ – $Sn_{Bi}^{/}$) + h_{VBM}^{+} or to ($Sn_{Bi}^{\bullet}$ – $Sn_{Bi}^{\times}$) + e_{CBM}^{-}), charge corrections were performed using the GKFO method for vertical defect transitions,[4] again with a 2/3 scaling of the point-charge correction energy. For calculations of the energy of inter-valence charge transfer (IVCT), corresponding to the optical transition ($Sn_{Bi}^{\bullet}$ – $Sn_{Bi}^{/}$) to ($Sn_{Bi}^{\times}$ – $Sn_{Bi}^{\times}$), the excited-state electron band occupancies were fixed to ($Sn_{Bi}^{\times}$ – $Sn_{Bi}^{\times}$) and a static total energy calculation was performed.

To determine the chemical potential limits of the system, structural relaxation and calculation of total energies were performed for the relevant competing phases and elemental references, using the same level of theory (HSE06+SOC) and plane-wave energy cutoff (350 eV), with a well-converged **k**-point mesh in each case. To ensure the equilibrium structure was used in

each case, all structures within 70 meV of the GGA DFT-predicted ground state (as provided by the Materials Project database; materialsproject.org) were considered for each material. In total, 40 competing and reference phases were calculated.

Bader charge density partitioning for the comparison of species' oxidation states was performed using the *bader* package as provided by the Henkelman group.[5]

All calculation data and analyses are provided in an online repository at doi.org/10.5281/zenodo.4780949.

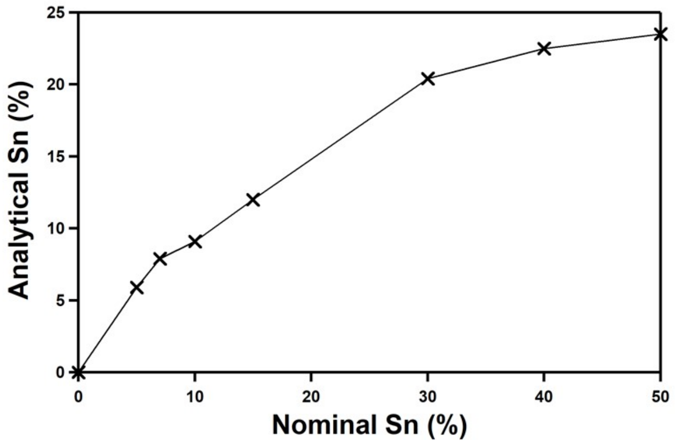

Figure S1: Analytical amounts of Sn within the $Cs_3Bi_2Br_9$ samples determined by XPS analysis as a function of the nominal concentration used in the synthesis.

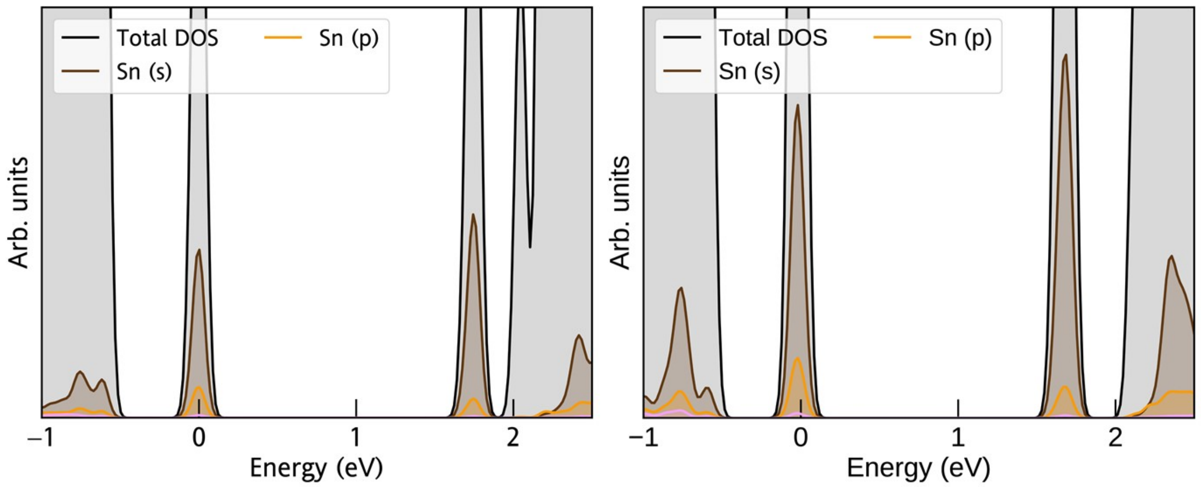

Figure S2. Orbital-projected electronic density of states for the optically-excited ($Sn_{Bi}^{\times}$ – $Sn_{Bi}^{\times}$) double-substitution complex in $Cs_3Bi_2Br_9$ for atomic Sn_{Bi} concentrations of 12.5% (left) and 25% (right). Position of lowest-energy unoccupied Sn_{Bi} state set to 0 eV (which in this case is located below the highest occupied (Sn_{Bi}) electronic state at ~1.75 eV).

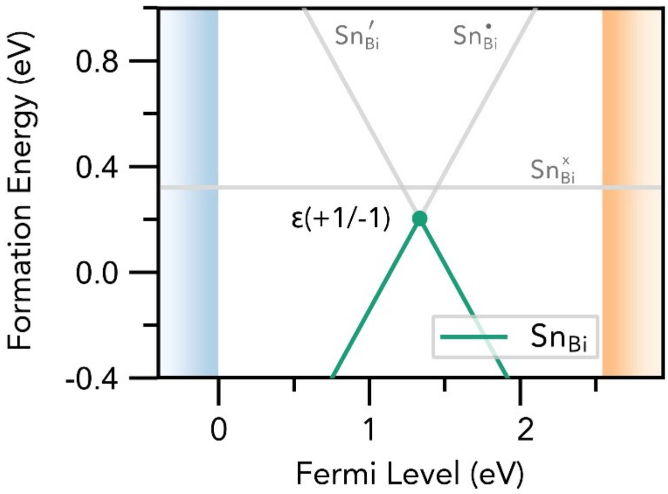

Figure S3. Defect formation energy diagram for the isolated Sn_{Bi} substitution in $Cs_3Bi_2Br_9$ under Sn-rich conditions. Valence band in blue, conduction band in orange. Equilibrium charge state highlighted in green.

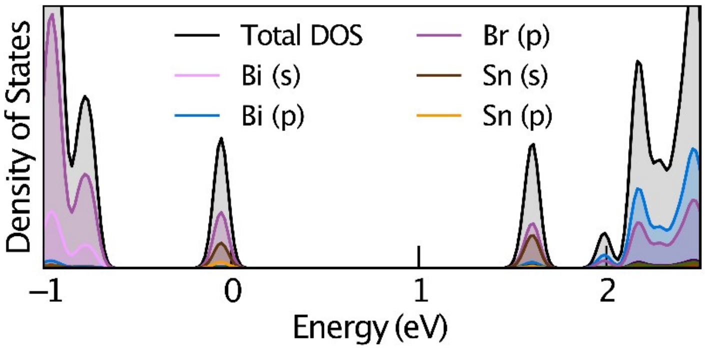

Figure S4. Orbital-projected electronic density of states for the ($Sn_{Bi}^{\bullet}$ – $Sn_{Bi}^{/}$) double-substitution complex in $Cs_3Bi_2Br_9$. Position of zero occupancy set to 0 eV.

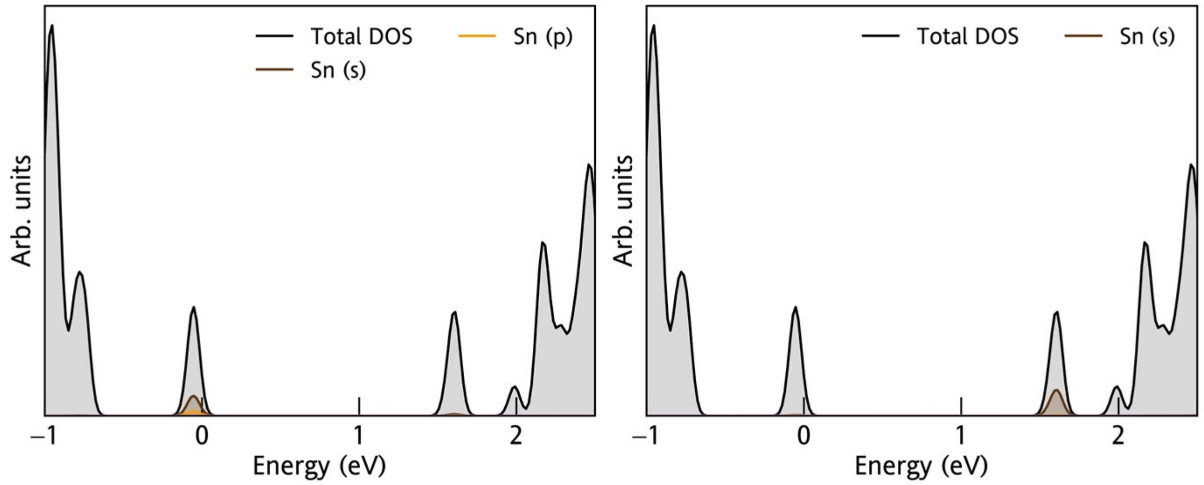

Figure S5. Site-projected electronic density of states for the $Sn_{Bi}^{/}$ (left) and $Sn_{Bi}^{\bullet}$ (right) sites in the ($Sn_{Bi}^{\bullet}$-$Sn_{Bi}^{/}$) double-substitution complex in $Cs_3Bi_2Br_9$. Position of zero occupancy set to 0 eV.

Crystal Orbital Hamilton Population (COHP) analysis involves partitioning the band-structure energy of a material, in this case Sn-doped $Cs_3Bi_2Br_9$, into a sum of pairwise atomic orbital interactions.[6] In this manner, COHP analysis allows the decomposition of the electronic density of states into bonding, nonbonding, and anti-bonding energy regions, as shown in Figure S6.

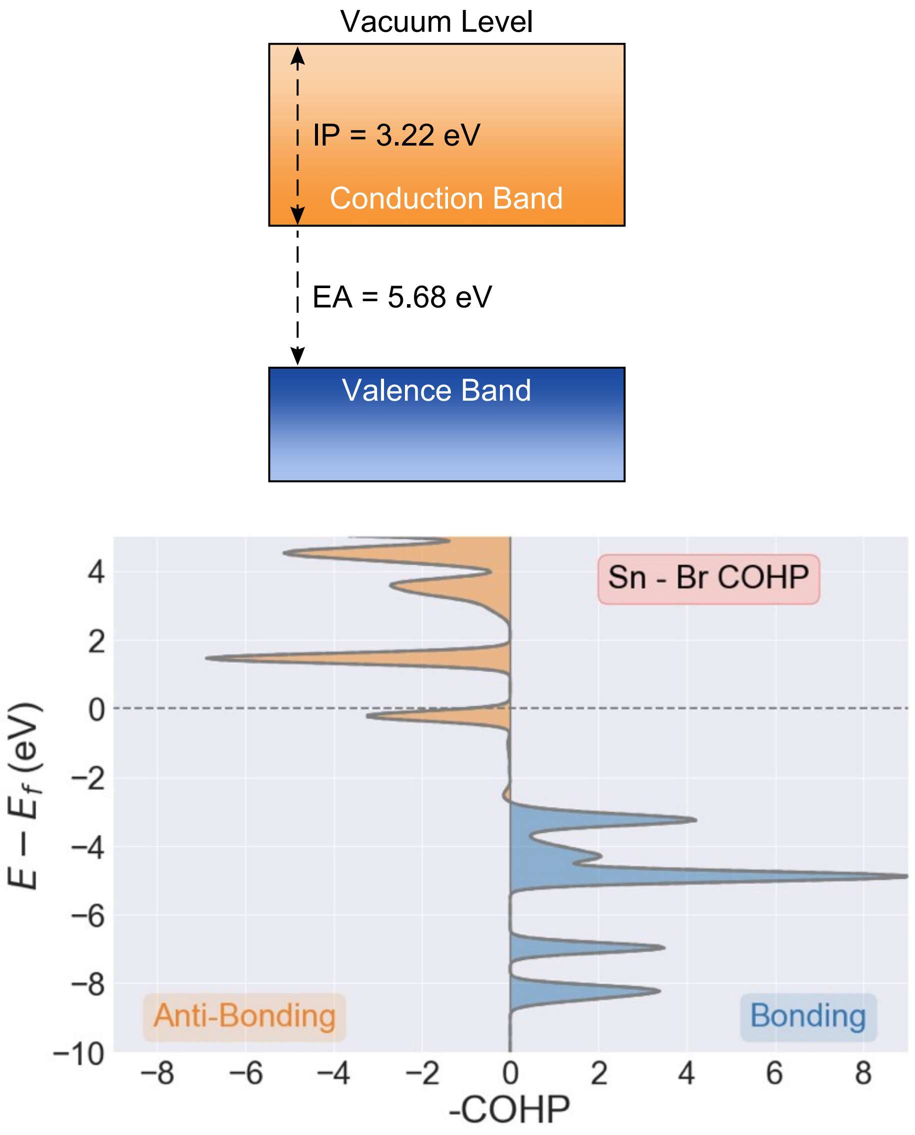

Figure S6. Calculated electronic band alignment of $Cs_3Bi_2Br_9$ (top), showing the ionisation potential (IP) and electron affinity (EA). (Bottom) Sn-Br atom-decomposed COHP(E) analysis of the electronic density of states in Sn-doped $Cs_3Bi_2Br_9$. Negative COHP values (blue) indicate energy-lowering, bonding-type interactions, while positive values (orange) indicate anti- bonding character.

In the COHP plot in Figure S6, the peak just below the Fermi level corresponds to the occupied Sn(II)-Br state in the bandgap (Figure 3d), while the peak ~1.5 eV above this corresponds to the unoccupied Sn(IV)-Br state. We see that, while the lower-energy Sn(II)-Br state is indeed overall anti-bonding in nature, it is significantly less so than the Sn(IV)-Br state. This is facilitated by the additional hybridisation of Sn 5*p* and strong electron-phonon coupling (allowing substantial distortion of the local environment), simultaneously reducing the anti-

bonding character of the Sn(II) 'lone-pair' state and firmly splitting the Sn-Br levels in the band gap.

Dielectric Constant

Due to the extremely-shallow, anharmonic barrier between the two structural polymorphs of $Cs_3Bi_2Br_9$ (Δ(Formation Energy) ~ 5 meV), corresponding to a pseudo-Jahn-Teller distortion of the $BiBr_6$ octahedra,[7] standard Density Functional Perturbation Theory (DFPT) or finite-difference calculations[8] of the ionic dielectric constant are not possible without in-depth phonon investigations, which are beyond the scope of this work. Hence, extrapolation of experimental values[9] for the dielectric constant (ε_0) were used to determine the room-temperature ionic dielectric response (ε_{Ionic}).

Table S1. Dielectric response of $Cs_3Bi_2Br_9$. Optical response (ε_{Optic}) calculated using HSE06+SOC (see Computational Methodology). Total dielectric constant at room-temperature (ε_0) extrapolated from literature values.[9] Ionic response (ε_{Ionic}) determined using the relation $\varepsilon_0 = \varepsilon_{Optic} + \varepsilon_{Ionic}$.

ε_{Optic} (ε_∞)	$\varepsilon_{Ionic\ (a,b)}$	$\varepsilon_{Ionic\ (c)}$	$\varepsilon_{0\ (a,b)}$[15]	$\varepsilon_{0\ (c)}$[15]
3.89	5.50	7.85	**9.39**	**11.74**

Instability of the Singly-Charged (Sn_{Bi} – Sn_{Bi}) Defects

Due to the positioning of the electronic Sn_{Bi} defect states in the gap (Figure S4): the 'acceptor level' position ΔE_A, corresponding to the energetic separation of the VBM and the higher Sn *s* – Br *p* level (i.e. $Sn_{Bi}^{\bullet}$, unoccupied in the ($Sn_{Bi}^{\bullet}$-$Sn_{Bi}^{/}$) neutral state), is approximately 2.14 eV, which is slightly greater than the 'donor level' position ΔE_D, corresponding to the energetic separation of the CBM and the lower Sn *s* – Br *p* level (i.e. $Sn_{Bi}^{/}$, occupied in the neutral state), which is approx. 2.04 eV. Hence, the +1 charge state (i.e. hole in donor level) is stabilised relative to the -1 charge state (i.e. electron in acceptor level), because $\Delta E_D < \Delta E_A$.

The same is true for the +2 and -2 charge states. $\Delta E_D < \Delta E_A$ and the +2 charge state is doubly-stabilised relative to the -2 charge state. Thus, the instability of the -1 charge state, relative to the neutral/-2 charge states, is slightly lower than that of the +1 charge state, relative to the neutral/+2 charge states.

This can be seen from the positions of the Sn_{Bi} defect levels in the gap in the DOSs for the neutral (Figures S4 and S5) and doubly-charged cases (in all cases, greater separation between VBM and acceptor levels than CBM and donor levels).

Chemical potential analysis establishes the following stability map (Figure S7) for $Cs_3Bi_2Br_9$, with CsBr and $BiBr_3$ determined as the most relevant competing phases.

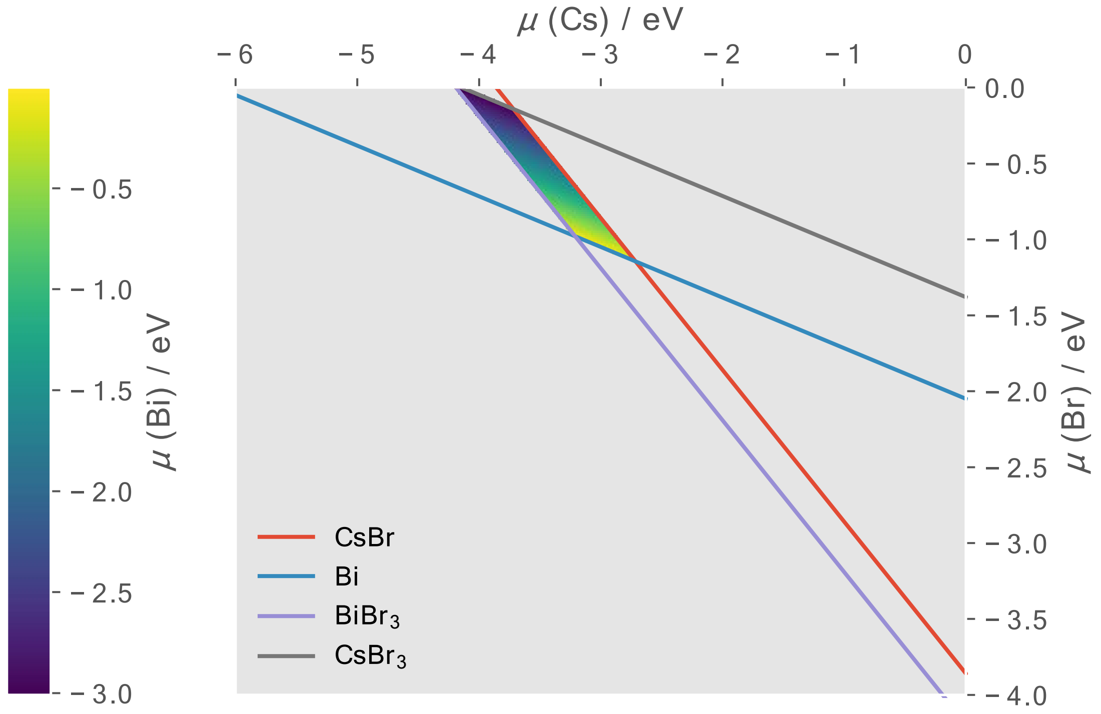

Figure S7. Stability map of the $Cs_3Bi_2Br_9$ ternary system, with the chemical potential of Bi as the dependent variable.

Both 12.5% and 25% Sn-doped $Cs_3Bi_2Br_9$ were found to be thermodynamically stable under Sn-rich conditions. The stability plots for the doped materials are shown Figure S8.

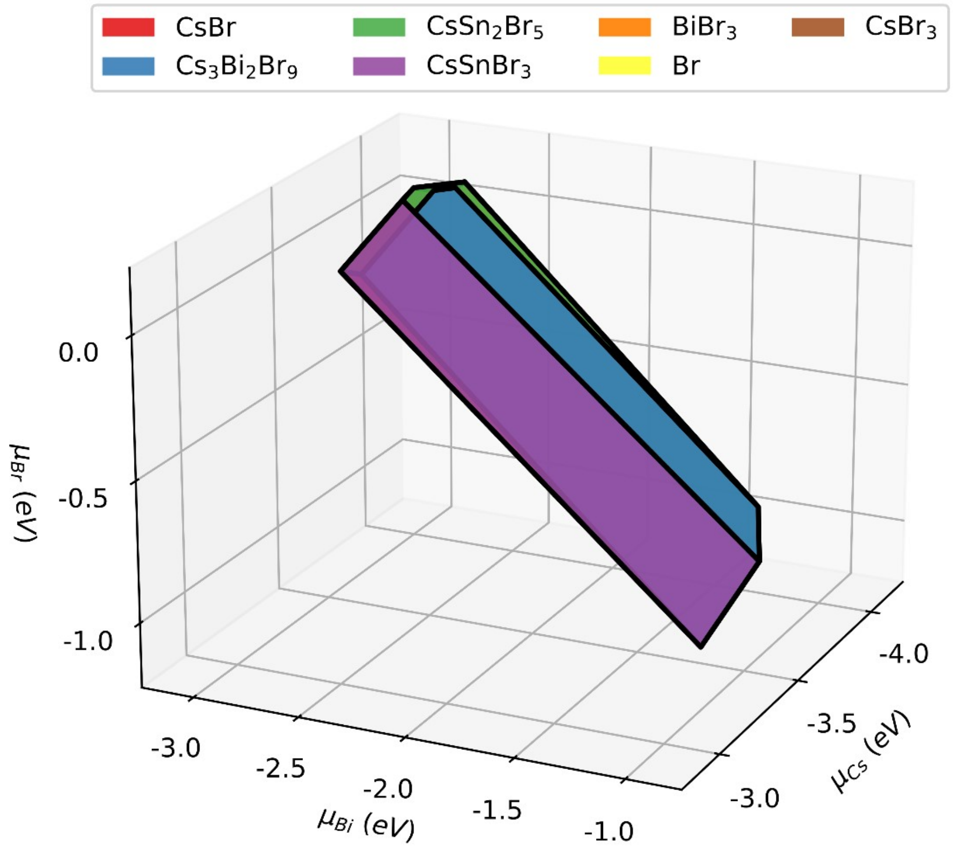

Figure S8. Stability map of the Sn: $Cs_3Bi_2Br_9$ quaternary system, with the chemical potential of Sn as the dependent variable.

At the Sn-rich limit, the competing phases are $CsSn_2Br_5$ and $CsSnBr_3$.

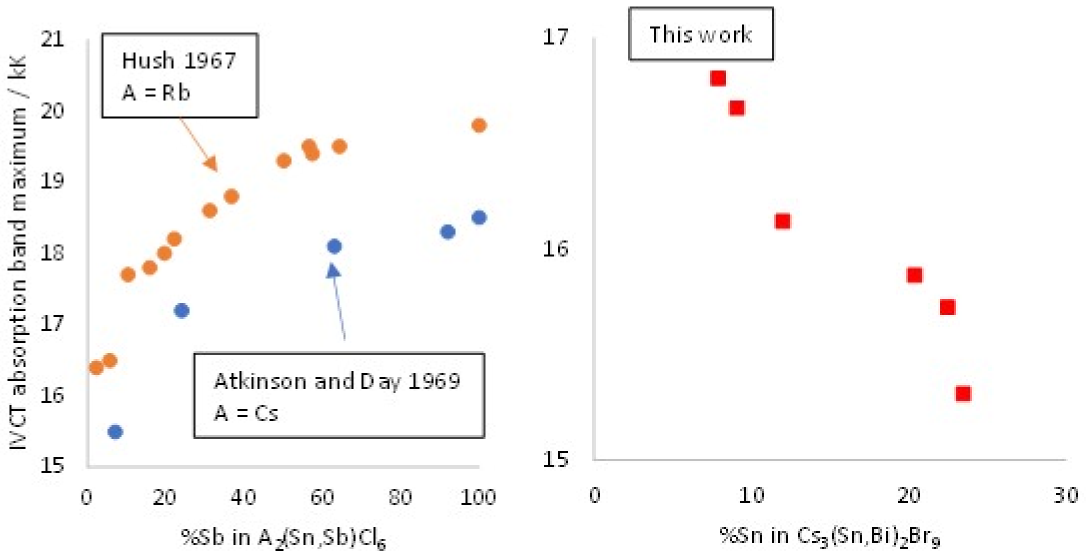

Figure S9. Variation in the intervalence absorption band maxima in previously reported A_2BX_6 structures (left) and $A_3B_2X_9$ structures reported in this work (right). The difference in behaviour is attributed to the defect motif formed.

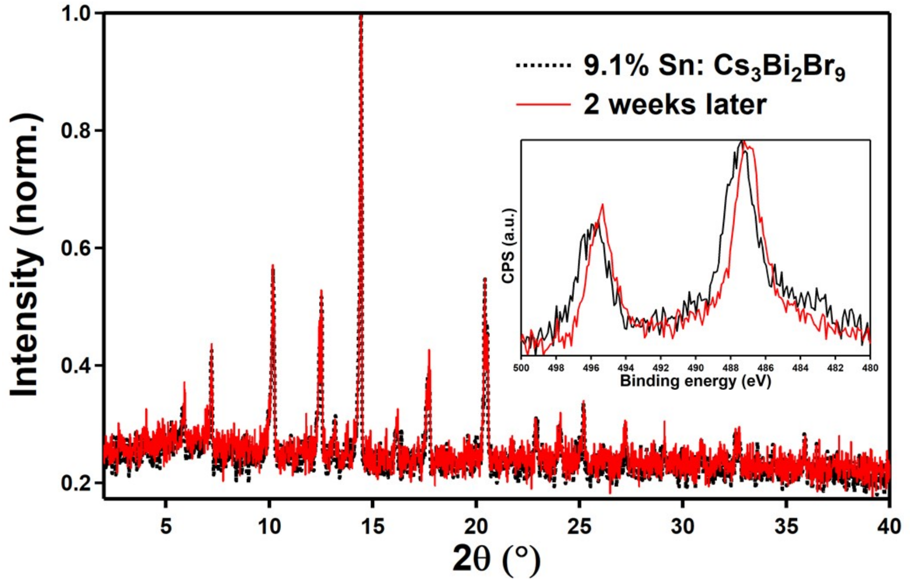

Figure S10. XRD patterns and, inset, XPS spectra for a representative Sn-doped $Cs_3Bi_2Br_9$ sample immediately after synthesis (black traces) and after storage in air for 2 weeks (red traces).

Table S2. Acquisition and processing parameters used for the NMR spectra presented in the main article.

^{133}Cs spectra

Composition	Figure	^{133}Cs T_1 [s]	Recycle delay [s]	Number of scans	Acquisition time [h]	Lorentzian apodization [Hz]
$Cs_3Bi_2Br_9$ ("0% Sn")	2A	132	700	5	1	0
"12% Sn"		176 (Cs_2SnBr_6), 101 ($Cs_3Bi_2Br_9$)	900	4	1	0
"23.5% Sn"		59-63 (across the whole spectrum)	350	4	0.4	10

^{119}Sn spectra

Composition	Figure	Recycle delay [s]	Number of scans	Acquisition time [h]	Lorentzian apodization [Hz]
"12% Sn" (tin oxide/halide range, red)	2B	15	5014	21	2000
"12% Sn" (0D range, blue)		15	11297	47	1000
"23.5% Sn" (tin oxide/halide range, red)		60	5866	98	2000
"23.5% Sn" (0D range, blue)		60	1491	25	1000
"23.5% Sn" (3D range, not shown) – no signal detected		0.025	20159	0.1	10000

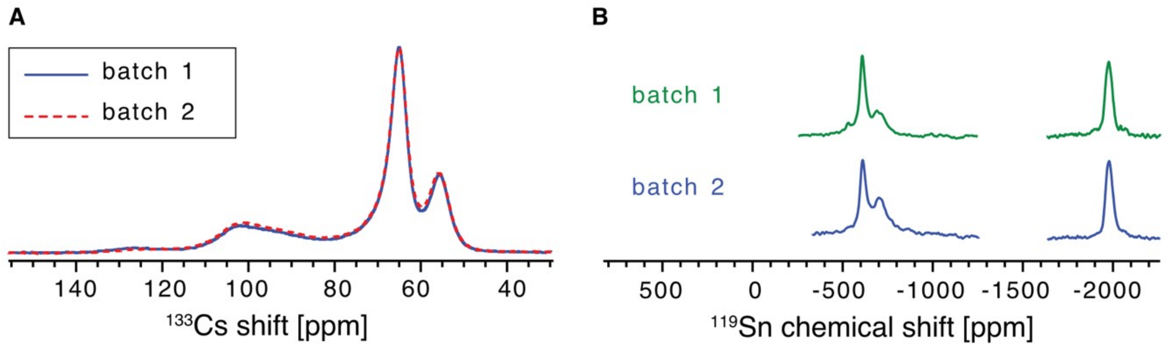

Figure S11. Comparison of solid-state NMR spectra of two separately synthesised batches of the 23.5% Sn-doped $Cs_3Bi_2Br_9$. A: quantitative ^{133}Cs NMR spectra. B: ^{119}Sn spectra. The ^{119}Sn spectra of batch 2 were recorded using a shorter recycle delay of 15 s, hence the small differences in lineshape).

Low-temperature PL

Figure S12 shows the high energy part of the low-temperature (10 K) photoluminescence spectra from Fig. 4 c. The undoped $Cs_3Bi_2Br_9$ exhibits a narrow free exciton peak at 2.67 eV, followed by a broader peak (~2.59 eV), resulting from recombination via defects located in the proximity of the surface.[10, 11] In the 9.1% Sn-doped sample, the free exciton peak of $Cs_3Bi_2Br_9$ persists over the broadened spectral emission, which is attributed to residual undoped $Cs_3Bi_2Br_9$. The overall photoluminescence intensity decreases approximately 20-fold. Further increase of Sn content to 23.5% results in significant redshift of the emission and disappearance of any feature characteristic of the undoped $Cs_3Bi_2Br_9$ host. The additional emission peaks observed upon Sn incorporation cannot be directly attributed to other Sn phases identified from NMR and are therefore attributed to radiative transitions originating from Sn-induced shallow defect states.

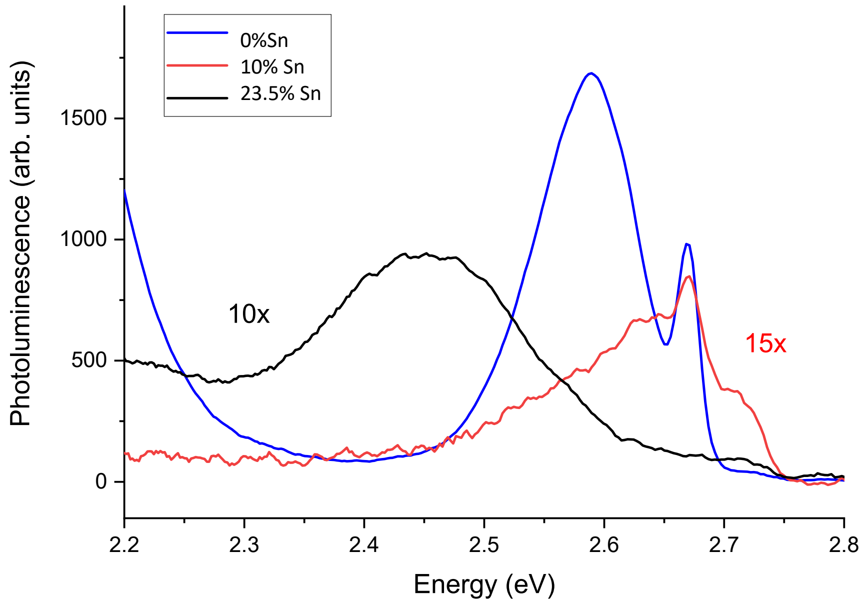

Figure S12. Low-temperature (10 K) steady-state photoluminescence spectra obtained for the undoped and Sn-doped $Cs_3Bi_2Br_9$. Intensities have been normalised by the multiplication factors given.

Thin Film Deposition and Characterisation

The as-prepared powders of undoped, 10% Sn-doped and 23.5% Sn-doped $Cs_3Bi_2Br_9$ were individually dissolved in a 4:1 (vol/vol) DMF/DMSO solvent mixture and stirred at 80°C for 2 hours. Upon filtration of the solutions with 0.2 µm PTFE syringe filters, thin films were prepared on glass substrates by spin coating at 3500 rpm for 50 s and then annealing at 200°C for 10 min. All processing of the perovskite solutions and subsequent fabrication of the thin films was performed under inert atmosphere. Three batches of films were made to ascertain reproducibility of the results. As shown in Figure S13, undoped and 10% Sn-doped thin films appear pale yellow, whilst the 23.5% Sn-doped sample is noticeably darker. Optical absorption spectra measured in transmission mode are shown in Figure S14.

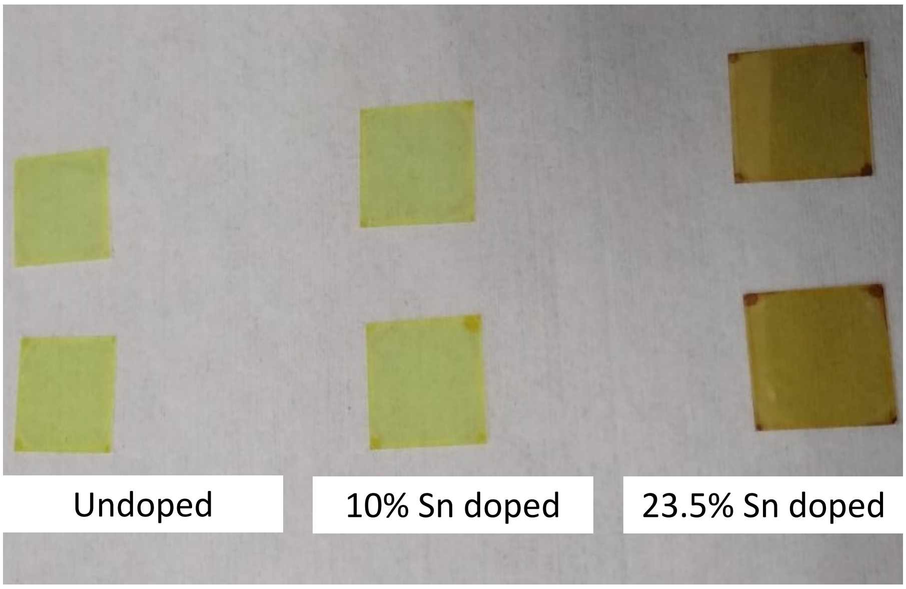

Figure S13. Photographs of films deposited from $Cs_3Bi_2Br_9$ and Sn-doped $Cs_3Bi_2Br_9$.

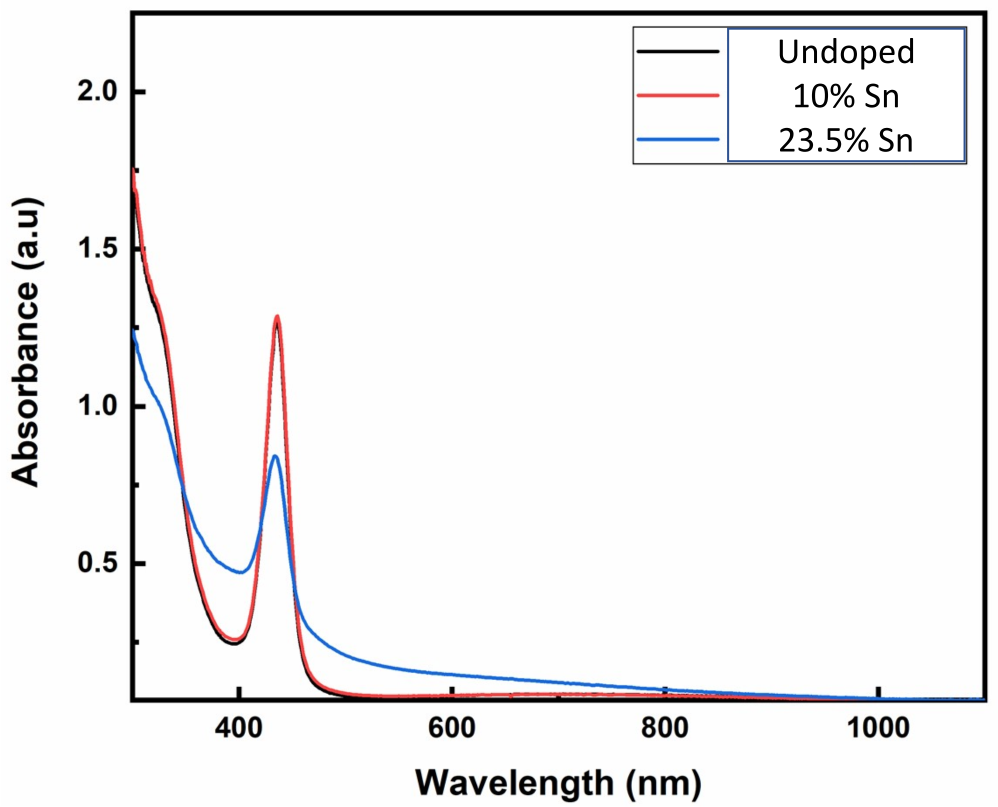

Figure S14. Absorption spectra of $Cs_3Bi_2Br_9$ and Sn-doped $Cs_3Bi_2Br_9$ thin films.

Quantification of the ^{119}Sn spectrum of the 23.5% Sn material

The material nominally contains 23.5% Sn. The quantitative ^{119}Sn spectrum shows that 28% of that is incorporated into $Cs_3Bi_2Br_9$: 0.235·0.28=0.0658. Since there are 2 Bi atoms per molecular unit, the formula of the doped materials is $Cs_3Bi_{1.87}Sn_{0.13}Br_9$.

We subsequently calculate the weight percent of each component. We first calculate the masses each of component (molecular weight · mol% from the quantitative spectrum):

SnO_2: 150.71 · 0.22 = 33 g

Cs_2SnBr_6: 863.95 · 0.51 = 441 g

$Cs_3Bi_{1.87}Sn_{0.13}Br_9$: 1524.08 · 0.28 / 0.13 = 3281 g

Sum = 3755 g

Finally, the weight percent is calculated as follows:

SnO_2: 33/3755 = 0.87 wt%

Cs_2SnBr_6: 431/3755 = 11.5 wt%

$Cs_3Bi_{1.87}Sn_{0.13}Br_9$: = 3281/3755 = 87.4 wt%

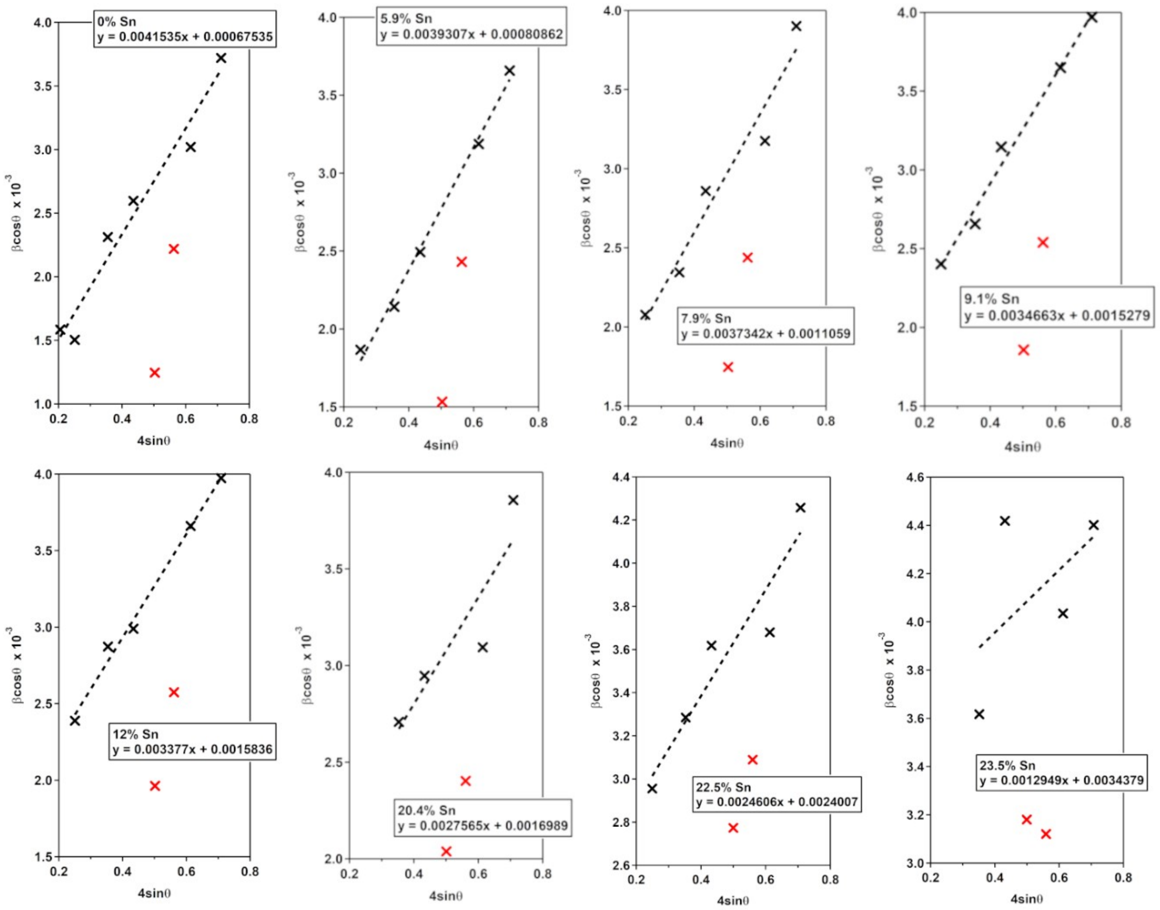

Figure S15. Williamson Hall plots of powder XRD data taken from Sn doped $Cs_3Bi_2Br_9$ with the analytical Sn contents shown.

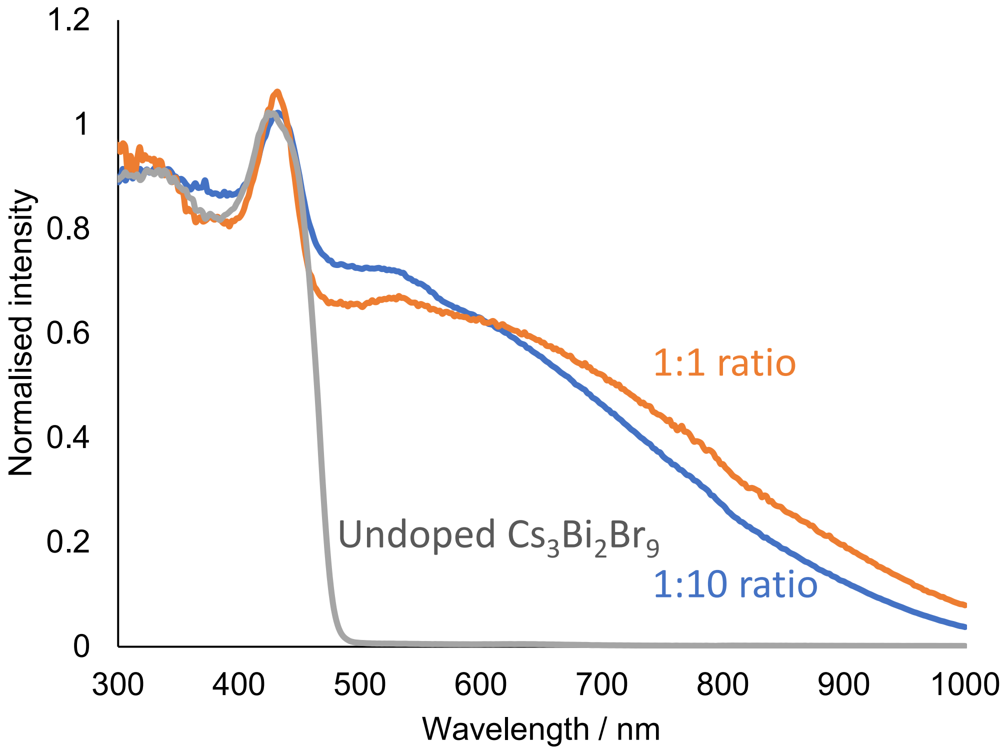

Figure S16. Diffuse reflectance spectra taken for undoped $Cs_3Bi_2Br_9$ (grey line) and Sn doped $Cs_3Bi_2Br_9$ using different sample : $BaSO_4$ weight ratios.

References

1. Lany, S.; Zunger, A., Assessment of correction methods for the band-gap problem and for finite-size effects in supercell defect calculations: Case studies for ZnO and GaAs. *Physical Review B* **2008,** *78* (23), 235104.
2. Lany, S.; Zunger, A., Accurate prediction of defect properties in density functional supercell calculations. *Modelling and Simulation in Materials Science and Engineering* **2009,** *17* (8), 084002.
3. Kavanagh, S. R.; Walsh, A.; Scanlon, D. O., Rapid Recombination by Cadmium Vacancies in CdTe. *ACS Energy Letters* **2021,** *6* (4), 1392-1398.
4. Gake, T.; Kumagai, Y.; Freysoldt, C.; Oba, F., Finite-size corrections for defect-involving vertical transitions in supercell calculations. *Physical Review B* **2020,** *101* (2), 020102.
5. Henkelman, G.; Arnaldsson, A.; Jónsson, H., A fast and robust algorithm for Bader decomposition of charge density. *Computational Materials Science* **2006,** *36* (3), 354-360.
6. Dronskowski, R.; Bloechl, P. E., Crystal orbital Hamilton populations (COHP): energy-resolved visualization of chemical bonding in solids based on density-functional calculations. *The Journal of Physical Chemistry* **1993,** *97* (33), 8617-8624.
7. Bass, K. K.; Estergreen, L.; Savory, C. N.; Buckeridge, J.; Scanlon, D. O.; Djurovich, P. I.; Bradforth, S. E.; Thompson, M. E.; Melot, B. C., Vibronic Structure in Room Temperature Photoluminescence of the Halide Perovskite Cs3Bi2Br9. *Inorganic Chemistry* **2017,** *56* (1), 42-45.
8. Gajdoš, M.; Hummer, K.; Kresse, G.; Furthmüller, J.; Bechstedt, F., Linear optical properties in the projector-augmented wave methodology. *Physical Review B* **2006,** *73* (4), 045112.

9. Yu, B.-B.; Liao, M.; Yang, J.; Chen, W.; Zhu, Y.; Zhang, X.; Duan, T.; Yao, W.; Wei, S.-H.; He, Z., Alloy-induced phase transition and enhanced photovoltaic performance: the case of Cs3Bi2I9−xBrx perovskite solar cells. *Journal of Materials Chemistry A* **2019,** *7* (15), 8818-8825.
10. Timmermans, C. W. M.; Blasse, G., ON THE LUMINESCENCE OF CS3BI2BR9 SINGLE-CRYSTALS. *Physica Status Solidi B-Basic Research* **1981,** *106* (2), 647-655.
11. Timmermans, C. W. M.; Blasse, G., THE LUMINESCENCE AND PHOTOCONDUCTIVITY OF CS3BI2BR9 SINGLE-CRYSTALS. *Journal of Luminescence* **1981,** *24-5* (NOV), 75-78.
